# Supplementary material for: The value of confidence: Confidence prediction errors drive value-based learning in the absence of external feedback
Source: PLoS Comput Biol. 2022 Oct 3;18(10):e1010580. doi: 10.1371/journal.pcbi.1010580 (PMC9560614; doi:10.1371/journal.pcbi.1010580)
Supplement: S1 Appendix — Table A. Mixed logistic regression on the dependent variable correct in phases 1 and 3. Performance increases significantly across phases with feedback (significant positive effect of trial_number). Table B. Mixed linear regression on the dependent variable confidence in phases 1 and 3. Confidence increases significantly across phases with feedback (significant positive effect of trial_number). Table C. Mixed logistic regression on the dependent variable correct in phase 2. Performance does not change significantly (non-significant effect of trial_number). Table D. Mixed linear regression on the dependent variable confidence in phase 2. Confidence increases significantly (significant positive effect of trial_number). Table E. Mixed logistic regression on the dependent variable consistent (coding whether a choice was consistent to the choice in the previous appearance of a CS pair) in phase 2. Consistency increases significantly with the number of appearances of a CS pair (significant positive effect of trial_pair_repeat_nr). Table F. Mixed linear regression on the dependent variable rating_change (subjective value rating post-phase-2 minus rating pre-phase-2). Ratings did not increase significantly with the objective value of the respective CS (no significant effect of value). Table G. Mixed linear regression on the dependent variable rating_change (subjective value rating post-phase-2 minus rating pre-phase-2). In comparison to the regression analysis in S6 Table, here we included the interaction of objective CS value (value) and the duration of phase 2 (block_ntrials_phase2). (PDF) [file pcbi.1010580.s001.pdf]

Appendix – Behavioral mixed model analyses

Table A. Mixed logistic regression on the dependent variable *correct* in phases 1 and 3. Performance increases significantly across phases with feedback (significant positive effect of *trial_number*).

DV: correct	Coef.	Std.Err.	z	P> |z|	[0.025	0.975]
Intercept	-0.322	0.284	-1.136	0.256	-0.878	0.234
block_difficulty	-0.064	0.029	-2.198	0.028	-0.121	-0.007
block_value_level	-0.559	0.014	-38.658	0.000	-0.588	-0.531
block_stimulus_type	0.141	0.074	1.905	0.057	-0.004	0.286
block_ntrials_phase1	-0.016	0.011	-1.429	0.153	-0.037	0.006
block_ntrials_phase2	0.002	0.007	0.330	0.741	-0.011	0.016
trial_difficulty	-0.003	0.014	-0.256	0.798	-0.030	0.023
trial_value_chosen	0.551	0.011	48.780	0.000	0.529	0.573
trial_number	0.043	0.004	11.720	0.000	0.036	0.050

Table B. Mixed linear regression on the dependent variable *confidence* in phases 1 and 3. Confidence increases significantly across phases with feedback (significant positive effect of *trial_number*).

DV: confidence	Coef.	Std.Err.	z	P> |z|	[0.025	0.975]
Intercept	-0.001	0.064	-0.010	0.992	-0.125	0.124
block_difficulty	0.008	0.011	0.668	0.504	-0.015	0.030
block_value_level	-0.080	0.012	-6.821	0.000	-0.103	-0.057
block_stimulus_type	-0.001	0.010	-0.131	0.896	-0.022	0.019
block_ntrials_phase1	0.001	0.010	0.093	0.926	-0.019	0.021
block_ntrials_phase2	0.000	0.010	0.004	0.997	-0.020	0.020
trial_difficulty	0.032	0.008	3.989	0.000	0.016	0.048
trial_value_chosen	0.233	0.009	25.658	0.000	0.215	0.250
trial_number	0.427	0.006	68.202	0.000	0.415	0.440

Table C. Mixed logistic regression on the dependent variable *correct* in phase 2. Performance does not change significantly (non-significant effect of *trial_number*).

DV: correct	Coef.	Std.Err.	z	P> |z|	[0.025	0.975]
Intercept	-0.359	0.464	-0.774	0.439	-1.269	0.551
block_difficulty	-0.032	0.046	-0.698	0.485	-0.122	0.058
block_value_level	-0.592	0.023	-25.211	0.000	-0.638	-0.546
block_stimulus_type	0.221	0.119	1.858	0.063	-0.012	0.455
block_ntrials_phase1	0.018	0.017	1.059	0.290	-0.016	0.052
block_ntrials_phase2	0.027	0.016	1.620	0.105	-0.006	0.059
trial_difficulty	0.022	0.023	0.985	0.325	-0.022	0.066
trial_value_chosen	0.572	0.019	30.541	0.000	0.535	0.608
trial_number	-0.005	0.013	-0.351	0.726	-0.030	0.021

Table D. Mixed linear regression on the dependent variable *confidence* in phase 2. Confidence increases significantly (significant positive effect of *trial_number*).

DV: confidence	Coef.	Std.Err.	z	P> |z|	[0.025	0.975]
Intercept	-0.001	0.080	-0.008	0.994	-0.157	0.156
block_difficulty	0.009	0.020	0.463	0.644	-0.030	0.049
block_value_level	-0.065	0.021	-3.132	0.002	-0.106	-0.024
block_stimulus_type	0.002	0.019	0.119	0.905	-0.036	0.040
block_ntrials_phase1	0.064	0.019	3.353	0.001	0.027	0.102
block_ntrials_phase2	-0.010	0.018	-0.576	0.565	-0.045	0.025
trial_difficulty	0.053	0.011	4.676	0.000	0.031	0.075
trial_value_chosen	0.282	0.013	21.399	0.000	0.256	0.308
trial_number	0.029	0.009	3.118	0.002	0.011	0.047

Table E. Mixed logistic regression on the dependent variable *consistent* (coding whether a choice was consistent to the choice in the previous appearance of a CS pair) in phase 2. Consistency increases significantly with the number of appearances of a CS pair (significant positive effect of *trial_pair_repeat_nr*).

DV: consistent	Coef.	Std.Err.	z	P> |z|	[0.025	0.975]
Intercept	2.082	0.974	2.138	0.033	0.173	3.990
block_difficulty	-0.109	0.080	-1.365	0.172	-0.265	0.047
block_value_level	-0.052	0.031	-1.659	0.097	-0.113	0.009
block_stimulus_type	0.142	0.212	0.670	0.503	-0.273	0.557
block_ntrials_phase1	-0.015	0.031	-0.489	0.625	-0.076	0.046
block_ntrials_phase2	-0.003	0.046	-0.076	0.940	-0.093	0.086
trial_difficulty	0.073	0.030	2.425	0.015	0.014	0.132
trial_value_chosen	0.081	0.020	4.144	0.000	0.043	0.120
trial_number	-0.043	0.030	-1.446	0.148	-0.101	0.015
trial_pair_repeat_nr	0.483	0.261	1.852	0.064	-0.028	0.994

Table F. Mixed linear regression on the dependent variable *rating_change* (subjective value rating post-phase-2 minus rating pre-phase-2). Ratings did not increase significantly with the objective value of the respective CS (no significant effect of *value*).

DV: rating_change	Coef.	Std.Err.	z	P> |z|	[0.025	0.975]
Intercept	0.000	0.041	0.009	0.993	-0.079	0.080
block_difficulty	-0.021	0.039	-0.538	0.591	-0.097	0.055
block_value_level	-0.067	0.045	-1.490	0.136	-0.155	0.021
block_stimulus_type	0.049	0.038	1.305	0.192	-0.025	0.123
block_ntrials_phase1	-0.108	0.037	-2.917	0.004	-0.181	-0.035
block_ntrials_phase2	0.035	0.036	0.963	0.336	-0.036	0.105
value	0.026	0.039	0.679	0.497	-0.050	0.102

Table G. Mixed linear regression on the dependent variable *rating_change* (subjective value rating post-phase-2 minus rating pre-phase-2). In comparison to the regression analysis in Supplementary Table S6, here we included the interaction of objective CS value (*value*) and the duration of phase 2 (*block_ntrials_phase2*).

DV: rating_change	Coef.	Std.Err.	z	P> |z|	[0.025	0.975]
Intercept	0.003	0.040	0.073	0.942	-0.076	0.082
block_difficulty	-0.025	0.039	-0.646	0.518	-0.101	0.051
block_value_level	-0.066	0.045	-1.476	0.140	-0.154	0.022
block_stimulus_type	0.048	0.038	1.262	0.207	-0.026	0.121
block_ntrials_phase1	-0.109	0.037	-2.946	0.003	-0.181	-0.036
block_ntrials_phase2	0.038	0.036	1.054	0.292	-0.033	0.108
value	0.025	0.039	0.640	0.522	-0.051	0.100
value:block_ntrials_phase2	0.088	0.032	2.722	0.006	0.025	0.151

8
