# Supplementary figures and images for: The value of confidence: Confidence prediction errors drive value-based learning in the absence of external feedback
Source: PLoS Comput Biol. 2022 Oct 3;18(10):e1010580. doi: 10.1371/journal.pcbi.1010580 (PMC9560614; doi:10.1371/journal.pcbi.1010580)

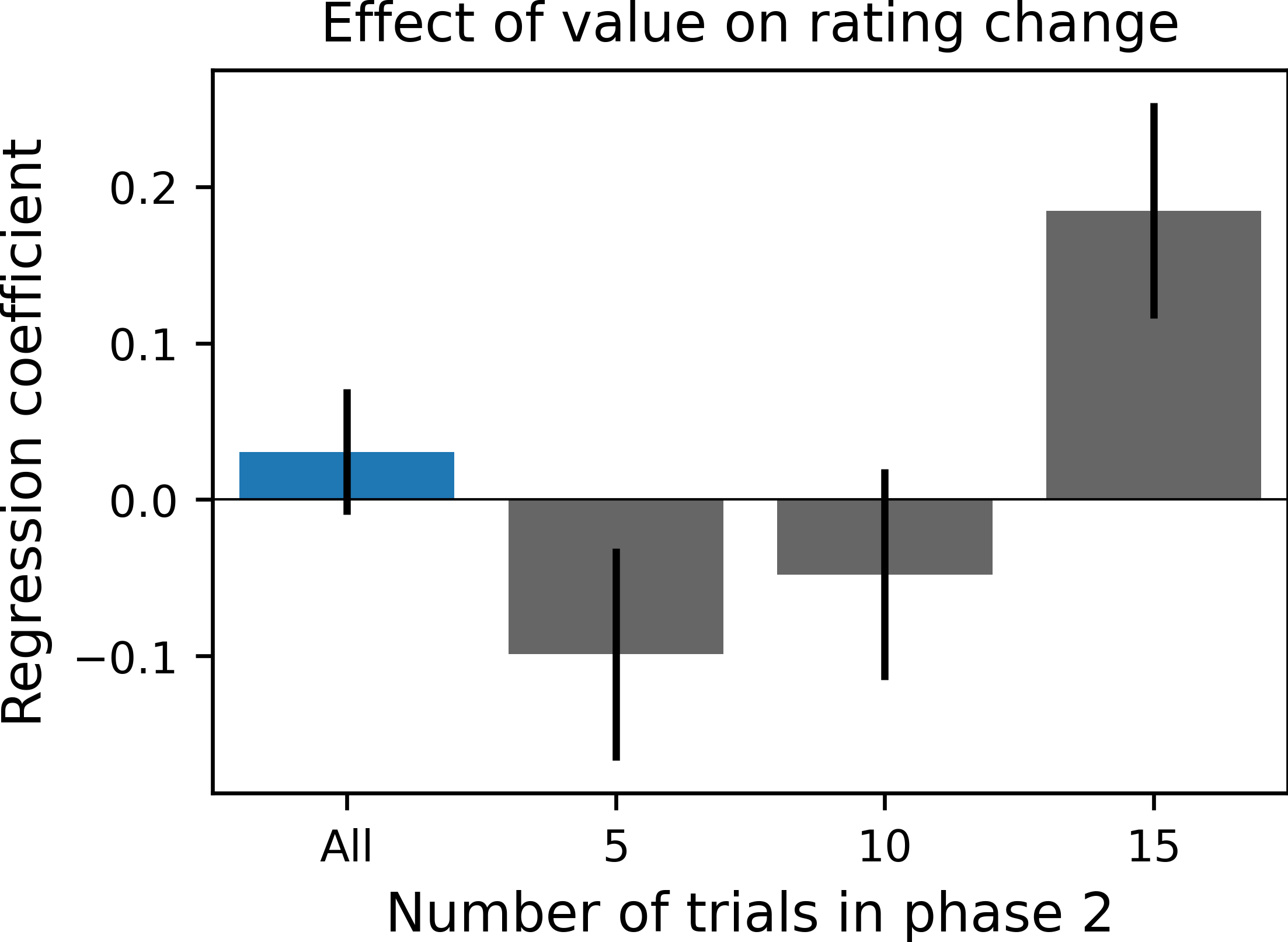

Supplement: S1 Fig — Regression coefficient for the effect of value on rating changes across varying durations of phase 2. (TIF) [file pcbi.1010580.s002.tif]

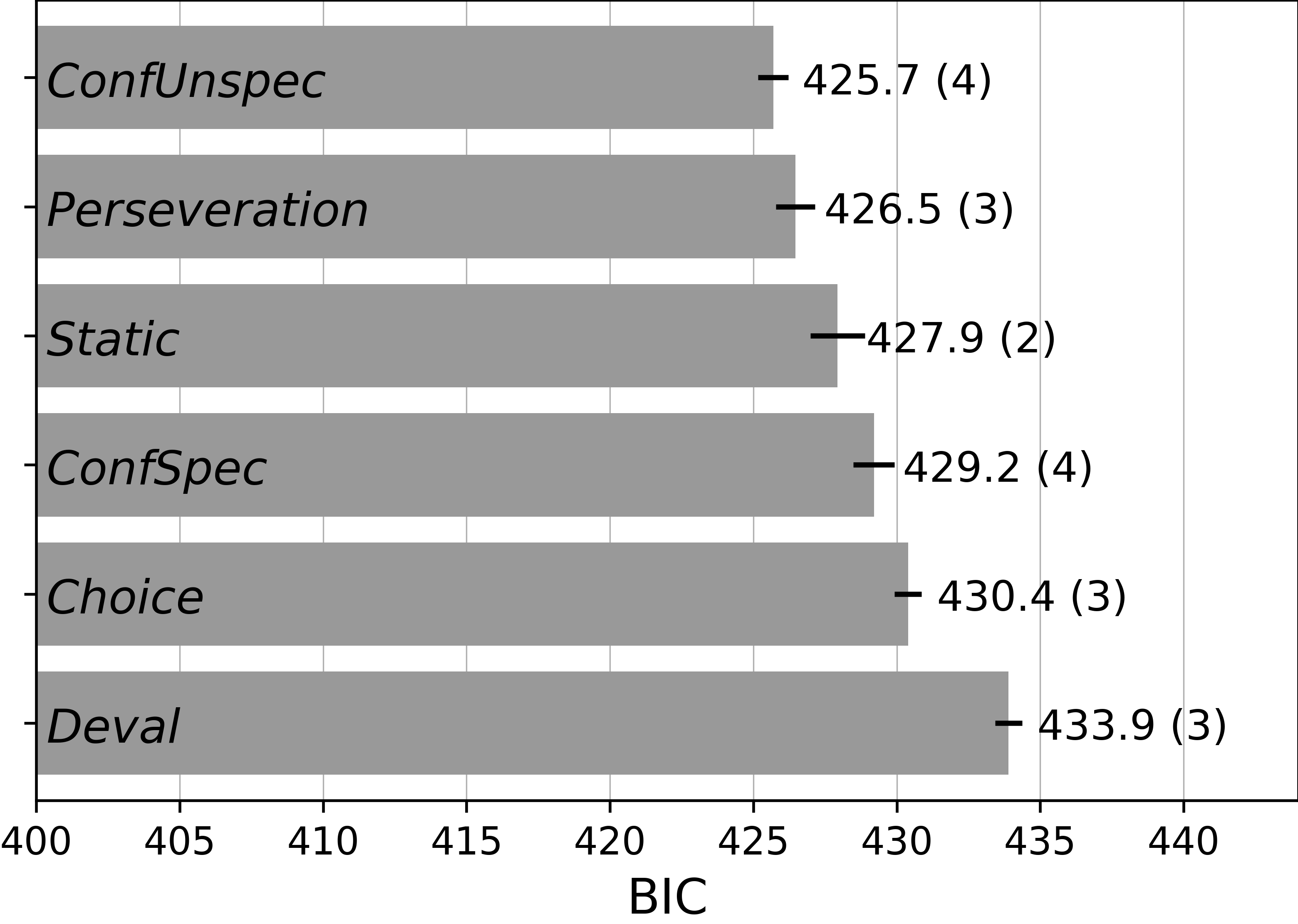

Supplement: S2 Fig — Average Bayesian information criterion with s.e.m. across participants for all computational models considered and ordered by model fit. The number of parameters is displayed in parentheses. In line with the Akaike information criterion (see Fig 4 in the manuscript), ConfUnspec is the winning model. (TIF) [file pcbi.1010580.s003.tif]

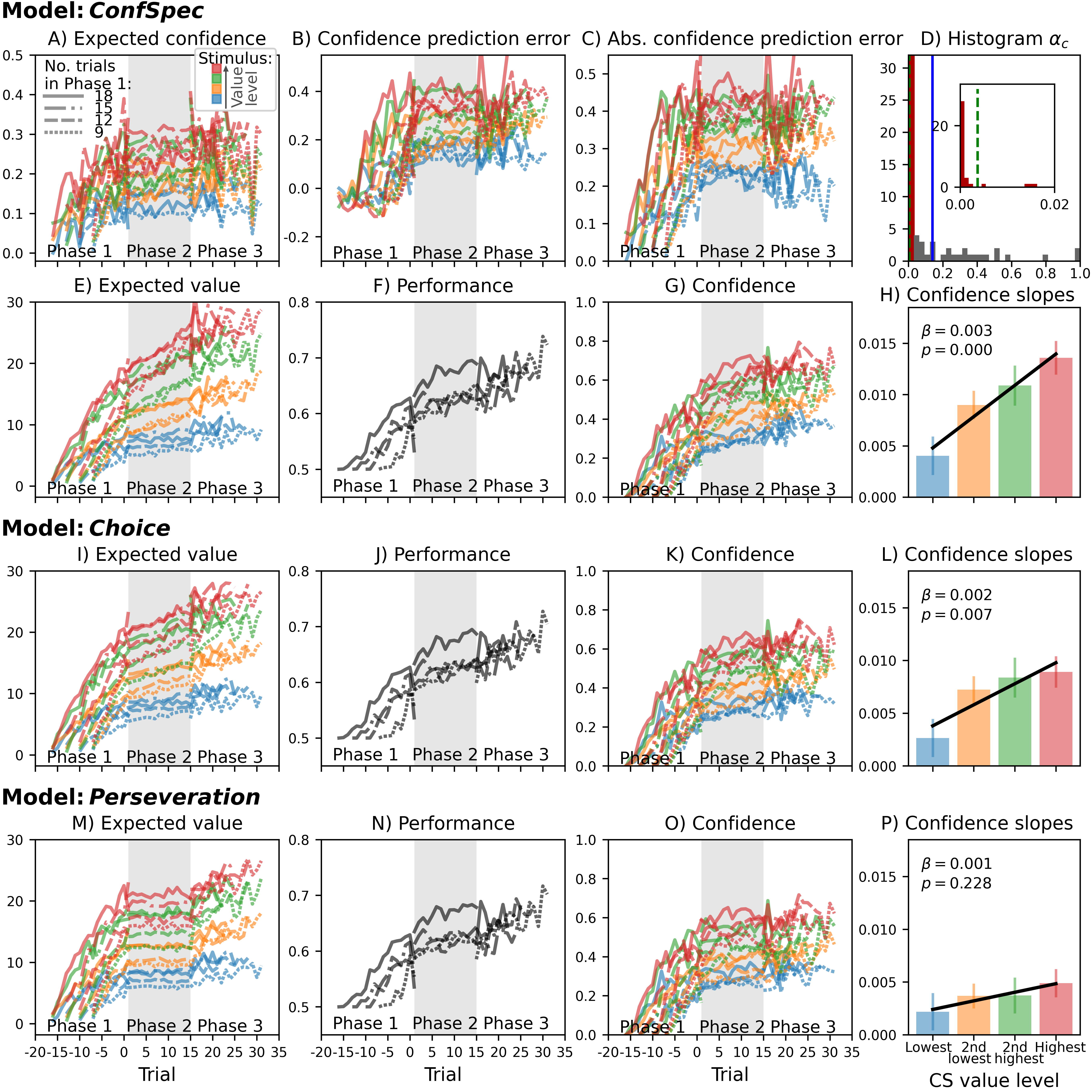

Supplement: S3 Fig — See Fig 5 for details. (TIF) [file pcbi.1010580.s004.tif]

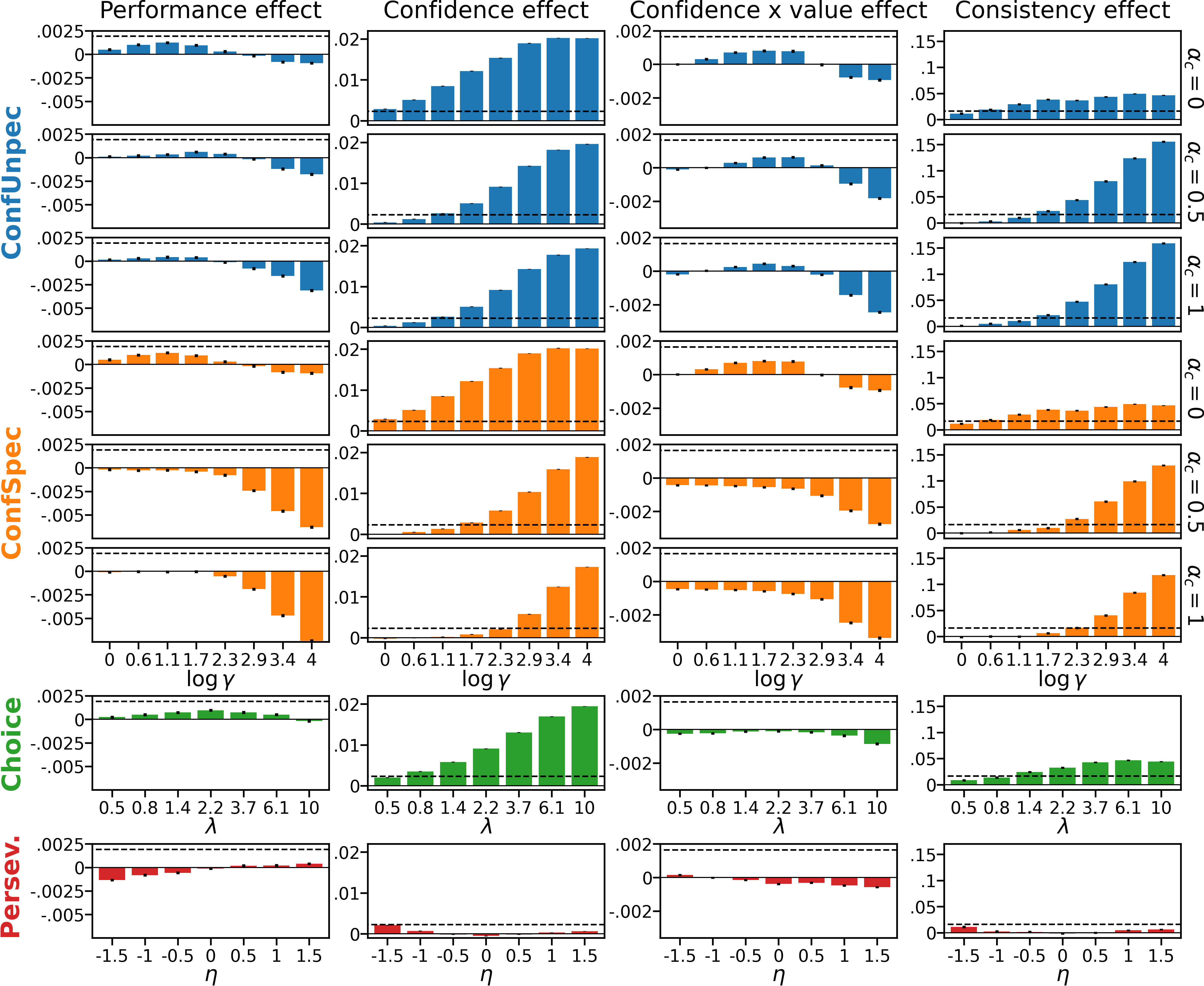

Supplement: S4 Fig — Model generative performance was assessed for the four best-performing models (ConfUnspec, ConfSpec, Choice, Perseveration). Model-based effects are shown as bar graphs, behavioral effects as dashed lines. Performance effect: model-based and behavioral graphs depict linear performance slopes in phase 2. Confidence effect: model-based and behavioral graphs depict linear confidence slopes in phase 2. Confidence x value effect: model-based and behavioral graphs depict slopes for the interaction of the Confidence effect and bandit value (cf. Fig 2C). Consistency effect: model-based and behavioral graphs depict the increase in choice consistency between the first and second occurrence of a choice pair versus the second and the third occurrence (cf. Fig 3A). Error bars for model-based effects indicate standard errors of the mean across 250 simulated subjects. (TIF) [file pcbi.1010580.s005.tif]

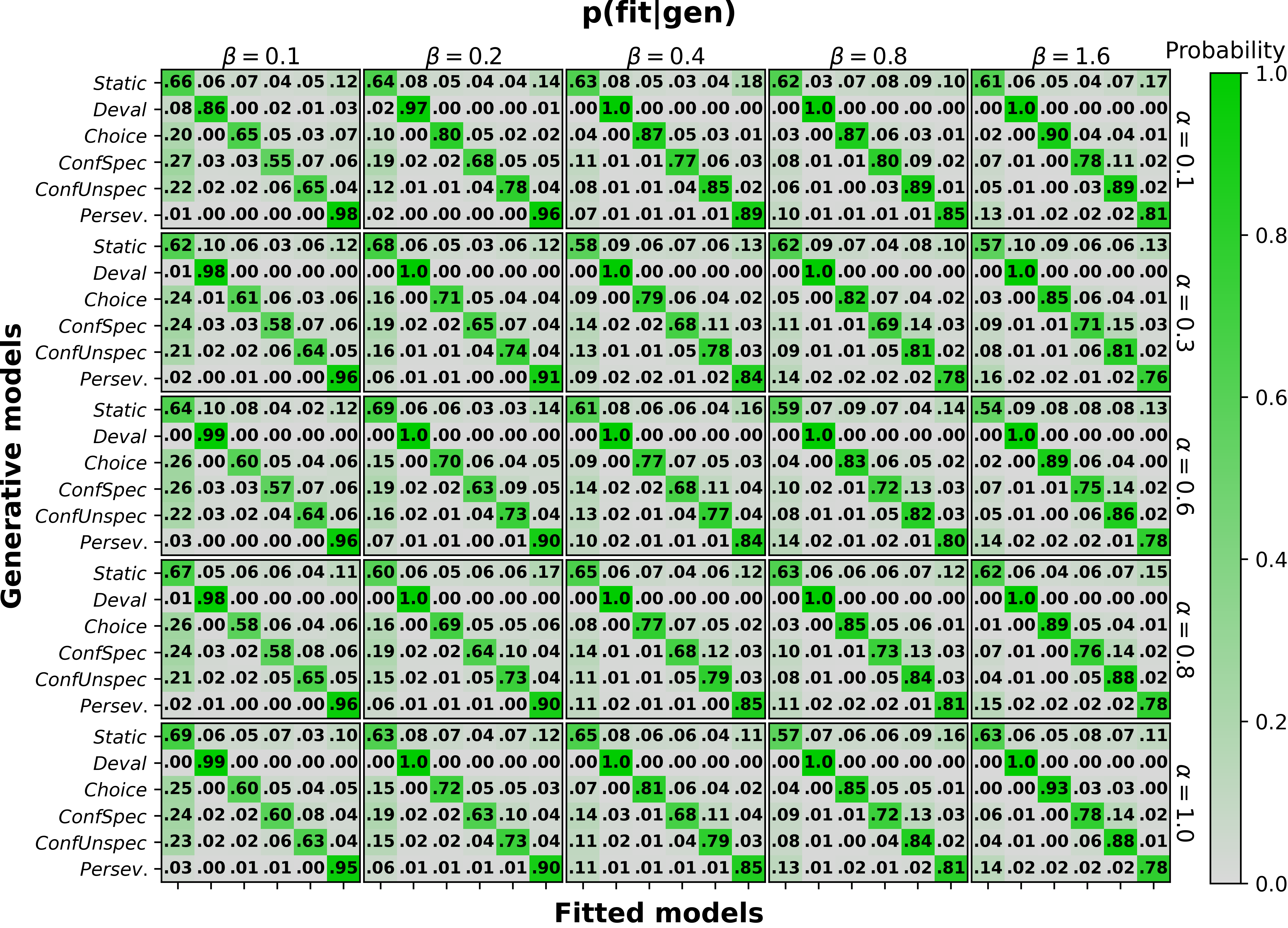

Supplement: S5 Fig — Rows represent generative models and each column within a row indicates the probability that a dataset was best fitted by a particular model. Note that the order of models is the same along both axes, but labels were omitted on the x-axis due to space constraints. (TIF) [file pcbi.1010580.s006.tif]

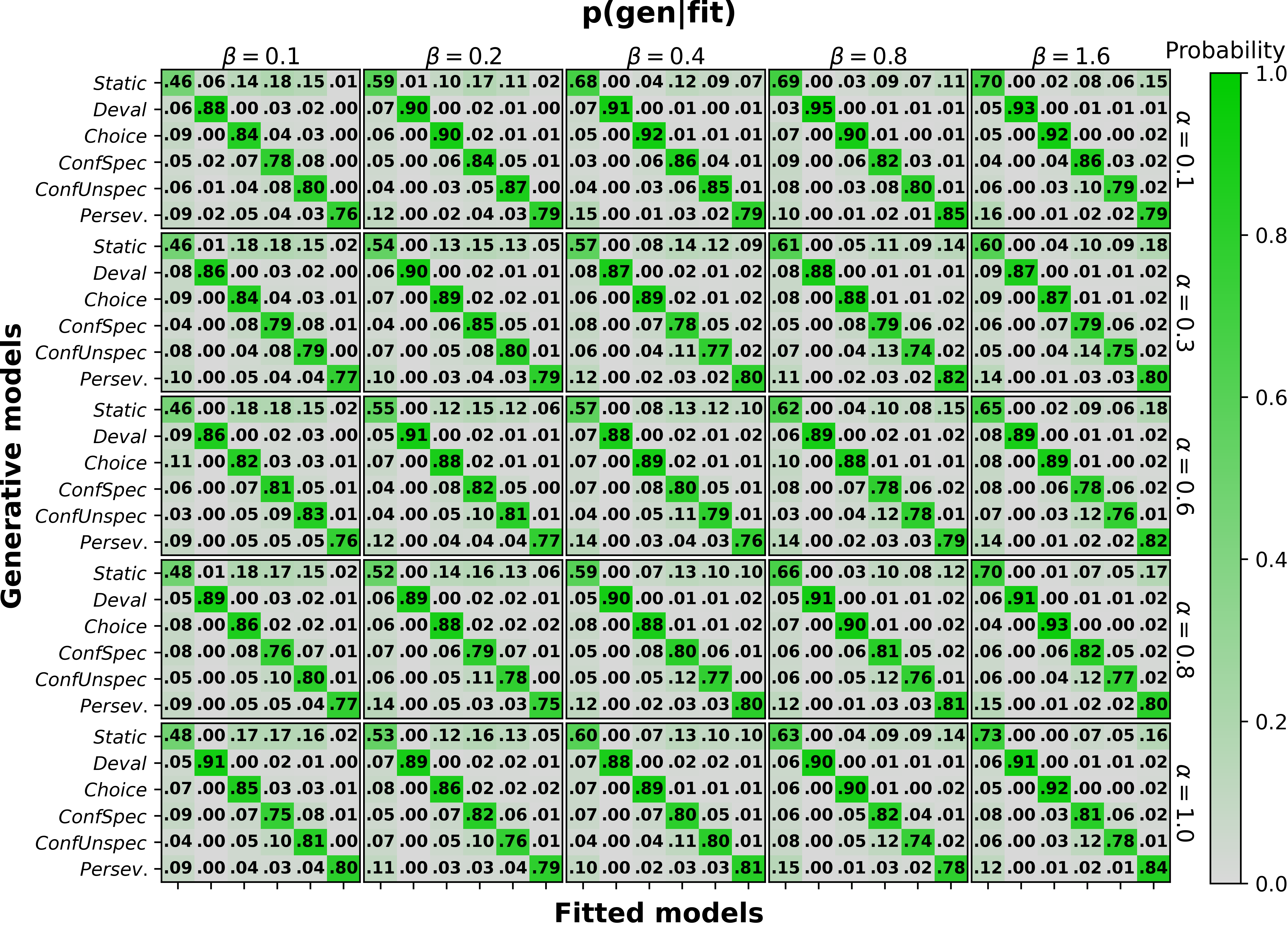

Supplement: S6 Fig — Rows represent the datasets in which the given model was best-fitting and each column within a row indicates the probability that the datasets were generated by a particular model. Note that the order of models is the same along both axes, but labels were omitted on the x-axis due to space constraints. (TIF) [file pcbi.1010580.s007.tif]

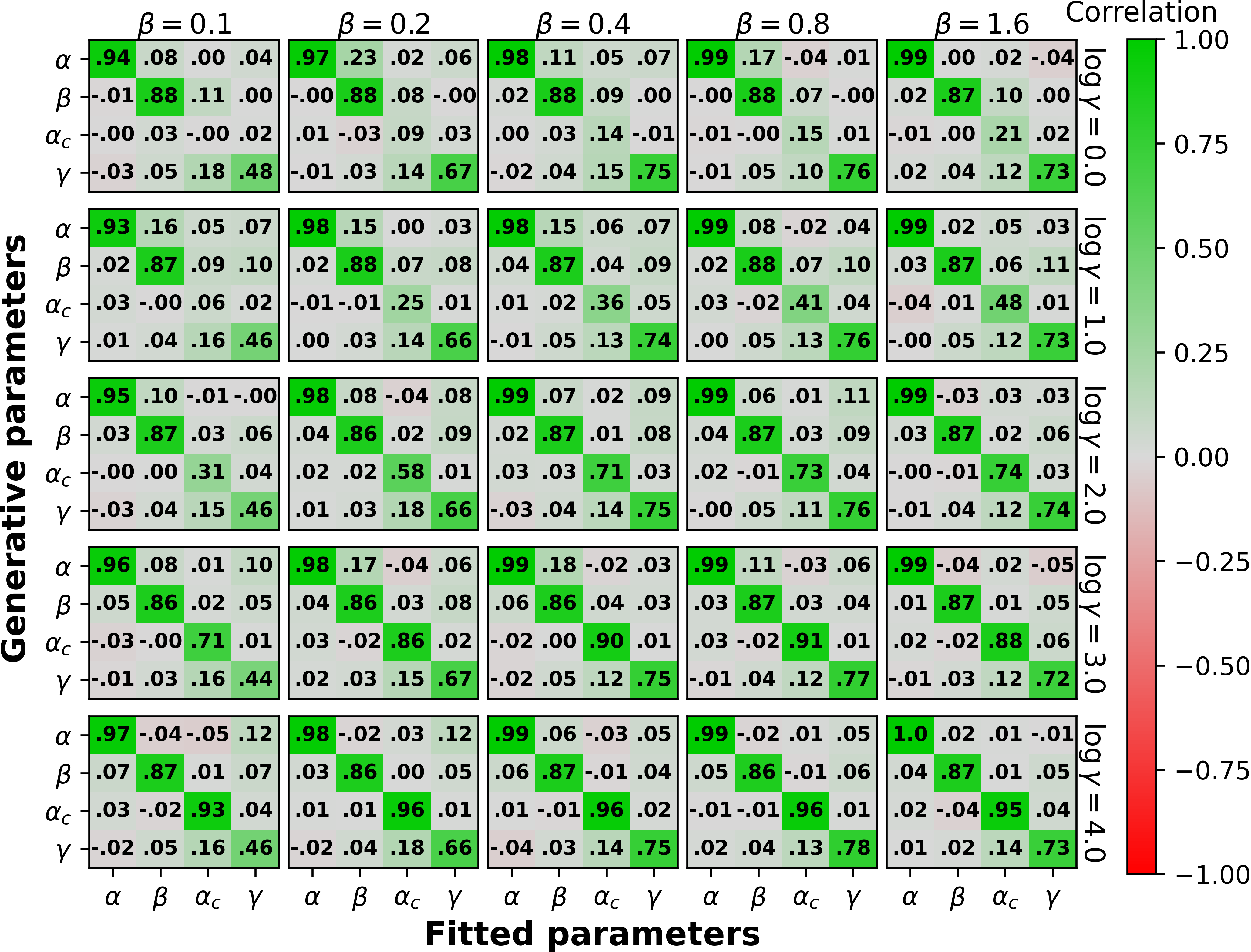

Supplement: S7 Fig — Pearson correlation matrices between generative parameters and fitted parameters in dependence of different settings for β and γ. The fixed β and γ values provided in the figure thus indicate the parameter values that were used for data generation in the construction of a recovery matrix. An exception is when β and γ were themselves varied–in these cases, the indicated values for β and γ do not apply; instead, different columns constitute internal replications for the recovery of β, and different rows constitute internal replications for the recovery of γ. (TIF) [file pcbi.1010580.s008.tif]
