# Supplementary material for: The value of confidence: Confidence prediction errors drive value-based learning in the absence of external feedback
Source: PLoS Comput Biol. 2022 Oct 3;18(10):e1010580. doi: 10.1371/journal.pcbi.1010580 (PMC9560614; doi:10.1371/journal.pcbi.1010580)
Supplement: Attachment — Submitted filename: Ptasczynski_etal_Response_to_reviewers.pdf [file pcbi.1010580.s009.pdf]

Reviewer #1: In this study, Ptasczynski and colleagues examine whether confidence can act as a learning signal, in the form of internal feedback, when external feedback is absent – an important but so far overlooked aspect of learning, presumably relevant to many real-life scenarios. They demonstrate that participants can maintain a running average of expected confidence, that is updated similarly as in standard RL mechanisms, and critically that is independent of the stimulus. They also rule out that stimuli are simply devalued in the absence of feedback. Overall, I found the rationale solid, and the paper is well written and can be an important contribution to the field.

The computational models are described comprehensively. Although it is definitely a strength that several computational models are included in the comparison + the best-fitting model variable trajectories are presented, we would like to see behavioral simulations of at least one alternative model, to validate the specificity of the mechanism at play.

Besides this main comment, I develop below by order of importance a number of comments to clarify some of the claims of the paper.

>> We would like to thank the reviewer for their thorough comments, which had a substantial impact on the manuscript.

Main comment

It is interesting that the authors have included behavioral predictions alongside their model-based approach, but we need more to validate the winning model:

- Could the authors provide behavioral signature(s) such as how different the predictions would be for each or some of the five models at play? (guidance of Palminteri et al 2017 TICS and Wilson & Collins 2019 eLife). The authors have already fitted the models, and validate the winning model by comparing some of its dynamics (Fig 5) to those of participants (Fig 2). The key next step would be to similarly simulate the second-winning (at least) model to help us assess whether each of the models actually do make different qualitative predictions, and understand where the difference in AIC between models comes from.

>> We agree that it would be elucidating to demonstrate differential predictions of the compared models and how those compare to the behavioral data. First of all, it is worth putting to the record that two of the compared models make predictions that are clearly different from both the behavioral data and the winning model: the *Static* and the *Deval* model, which predict no changes or devaluation of subjective values in phases without feedback. Now, had we included only one of the self-reinforcement models (the winning one) into the manuscript, one could consider the 'job done'. In full honesty, the reason we included all three self-reinforcement models (*ConfUnspec*, *ConfSpec* and *Choice*) in the manuscript was not because they made clear differential behavioral predictions, but rather for

transparency and completeness reasons, i.e. including all three mechanisms that we conceived would generate self-reinforcing behavior and that we have tested.

We now performed a simulation analysis to assess the predictions of the three self-reinforcing models. To anticipate the results, we did not find a 'smoking gun' that would explain why the *ConfUnspec* model is the winning one among those. Nevertheless, we think that the analyses further serve to illustrate the model dynamics at work and in the process we found one additional clear model prediction that we also confirm in the behavioral data (though once again all three self-reinforcing models show this pattern).

In a first step, we now included the development of estimated (reward) values for all three self-reinforcing models in the manuscript (Figure 5A and Supplementary Figure S4). All three models exhibit a spreading of values such that (overall) more valuable CS also show a stronger increase across phase 2, as to be expected for a self-reinforcement process. However, no clear differences are visible between models. Since we have no trial-wise behavioral reports of subjective values to which the models could be compared to, we next turned to confidence for which we have reports. We had previously not computed confidence predictions for the models since confidence prediction errors were based on the actual behavioral reports, but we now computed model-based confidence predictions based on the choice probability for the chosen stimulus (scaled to the range 0-1). As expected from the spreading of values, we find that all three models predict increases in confidence across phase 2 (Figure 5F and Supplementary Figure S4), like for the behavioral data (Figure 2B; new: separated by CS value level). For convenience we have included all three new/updated figures below this response.

On closer inspection, the increase in confidence for each model is higher for more valuable items. We quantified this in terms of slopes, separately computed for each CS value level, which shows a significant positive main effect of value level. Interestingly, the same pattern is present for the behavioral data (new Figure 2C). While once again this analysis does not single out one of the models, the correspondence to the behavioral data provides additional evidence for a self-reinforcement mechanism as implemented by our models.

We refer to this new analysis in the Result section as follows (p. 8, l. 149-152):

> "The increase in confidence in phase 2 was dependent on the overall value level of the chosen CS. A linear model with phase 2 confidence slope as DV and value level as IV indicated a significant positive effect of value level (β=0.002, p=0.033). Thus, confidence slopes were on average higher for more valuable CS (Figure 2C)."

Finally, we now also included the latent confidence-based learning signals of the second-best model (*ConfSpec*) in Supplementary Figure 4A-C (cf. Figure 5B-D for the *ConfUnspec* model). A comparison of both models indeed indicates a difference in the dynamics of both models, which originates from overall lower confidence learning rates α_c in *ConfSpec* (Supplementary Figure 4D). This results in a slower build-up of expected confidence compared to *ConfUnspec* and, as a consequence, in higher

confidence prediction errors (both in terms of the average and the average *absolute* prediction errors). On a speculative note, the fact that expected confidence in the *ConfSpec* model is substantially lower than in the *ConfUnspec* model *and* in behavioral confidence could provide some indication as to why the *ConfSpec* model falls short of the *ConfUnspec* model in terms of fitting our data.

To reflect these new analyses in the manuscript, we added a larger subsection within our Result section on latent model dynamics (p. 19-20, l. 320-347):

> "How do the predictions of the *ConfUnspec* model compare to the two other self-reinforcing models, *ConfSpec* and *Choice*, and to the *Perseveration* model? To find out, we assessed the latent variables and metacognitive predictions of these models as well. The second-best model, *ConfSpec*, has a confidence-based mechanism as well. As shown in Supplementary Figure 4A, the latent variables of *ConfSpec* − expected value, expected confidence, confidence prediction error − show largely the same patterns as for *ConfUnspec*. The main difference is that the median confidence learning rates α_c are, on average, lower for *ConfSpec*, which leads to a slower build-up of expected confidence; this in turn results in larger average and absolute average prediction errors compared to the winning *ConfUnspec* model (cf. Figure 5C, D) and thus more erratic learning signals. The fact that the time course of expected confidence does not timely track actual confidence may be an indication that the *ConfSpec* is a less adequate fit to the data than *ConfUnspec*.
>
> In terms of behavioral predictions for subjective values, performance and confidence, the winning model *ConfUnspec* (Figure 5A,F,G) is largely indistinguishable from the models *ConfSpec* (Supplementary Figure 4A) and *Choice* (Supplementary Figure 4B). In particular, all three models predict a spreading of subjective values in phase 2, and as a consequence, an increase of confidence. As observed for the behavioral data and the winning *ConfUnspec* model, *ConfSpec* and *Choice* likewise predict that the increase of confidence in phases without feedback is value-dependent (Supplementary Figures 4H, L). In contrast, while the *Perseveration* model does not predict changes in confidence in phase 2, it is the only model that predicts a flat type 1 performance curve. The reason is that the *Perseveration* model leaves expected values unchanged and merely causes choices to become more consistent (the perseveration bias η is positive for all participants; cf. Table 2). On the other hand, the fact that the self-reinforcing models predict an increase in performance (Figure 5E and Supplementary Figures 4F,J) has to be acknowledged as an incorrect prediction of the self-reinforcing models. Overall, at this point it remains somewhat opaque as to why specifically the *ConfUnspec* model outperforms the two other self-reinforcing models and the *Perseveration* model."

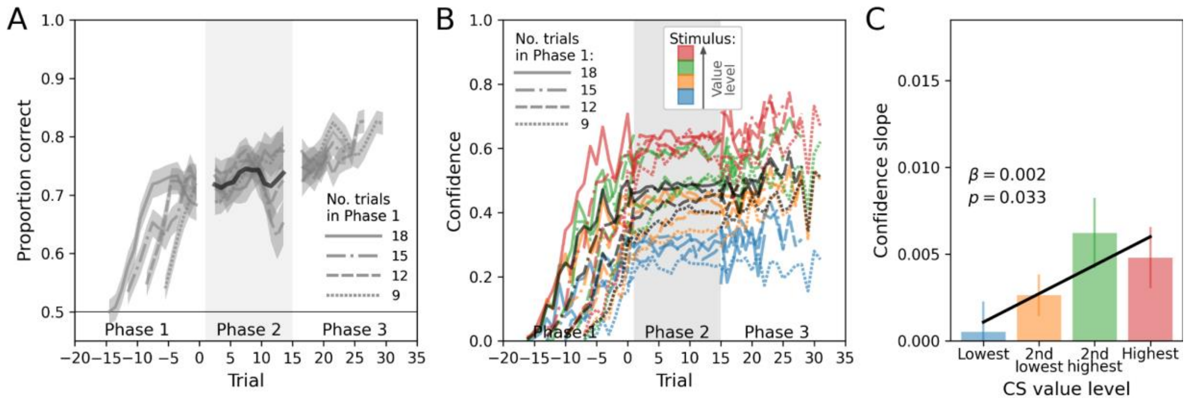

(Updated) Figure 2. Performance and confidence. Block-averaged time courses are separated according to the duration of phase 1 (9-18 trials) and aligned to the beginning of phase 2. Shaded areas indicate standard error of the mean. **(A)** Value-based learning. The accuracy of choices gradually increased across the phases with feedback (phases 1 and 3), indicating that participants successfully learned the task. **(B)** Confidence. Reported confidence (normalized to [0; 1]) likewise increases across the course of a block. Black lines indicate averages across CS value levels. **(C)** Confidence increases in phase 2 in dependence of the CS value level. The parameter estimate β and the p-value are based on a linear model with value level as IV and average confidence slope in phase 2 as DV.

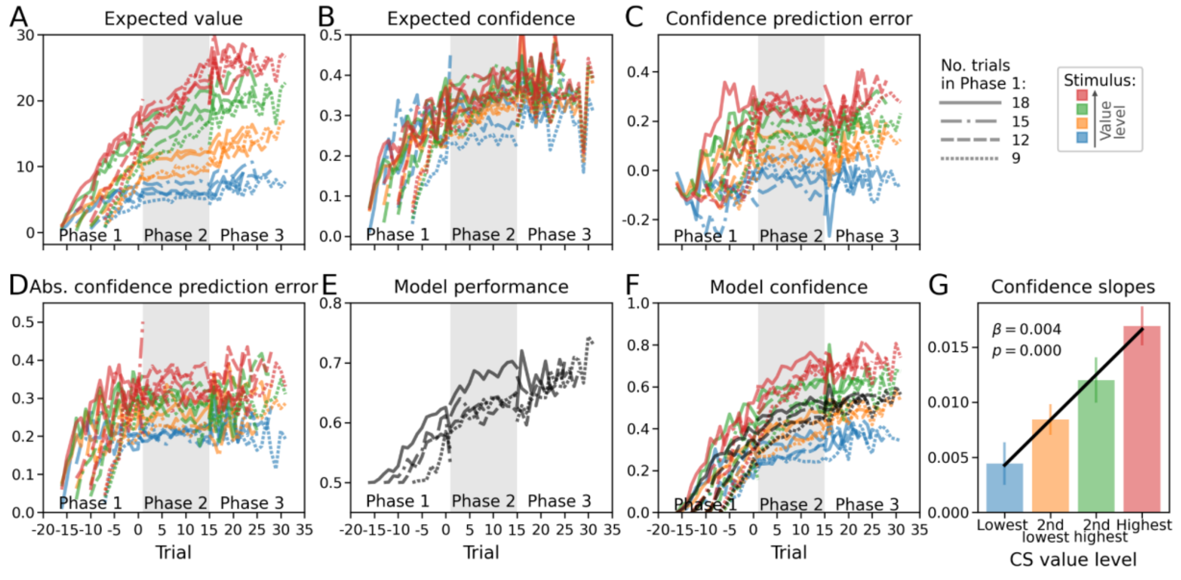

(Updated) Figure 5. Latent variables of model *ConfUnspec*. All time courses represent averages across blocks and subjects, split according to the duration of phase 1 (line styles) and the four CS value levels within a block (colors). **(A)** Expected values indicate the current beliefs about the value of each stimulus. **(B)** Expected confidence corresponds to an integration of past confidence experiences using a Rescorla-Wagner-type learning rule. **(C)** Confidence prediction errors indicate the deviation of a momentary confidence experience from expected confidence. **(D)** Absolute

confidence prediction errors. **(E)** Model performance: proportion correct responses for simulated model choices. **(F)** Model confidence is computed based on the choice probability for the chosen CS (normalized to the range 0-1). Black lines indicate averages across value levels. **(G)** Confidence slopes in phase 2 in dependence of the CS value level.

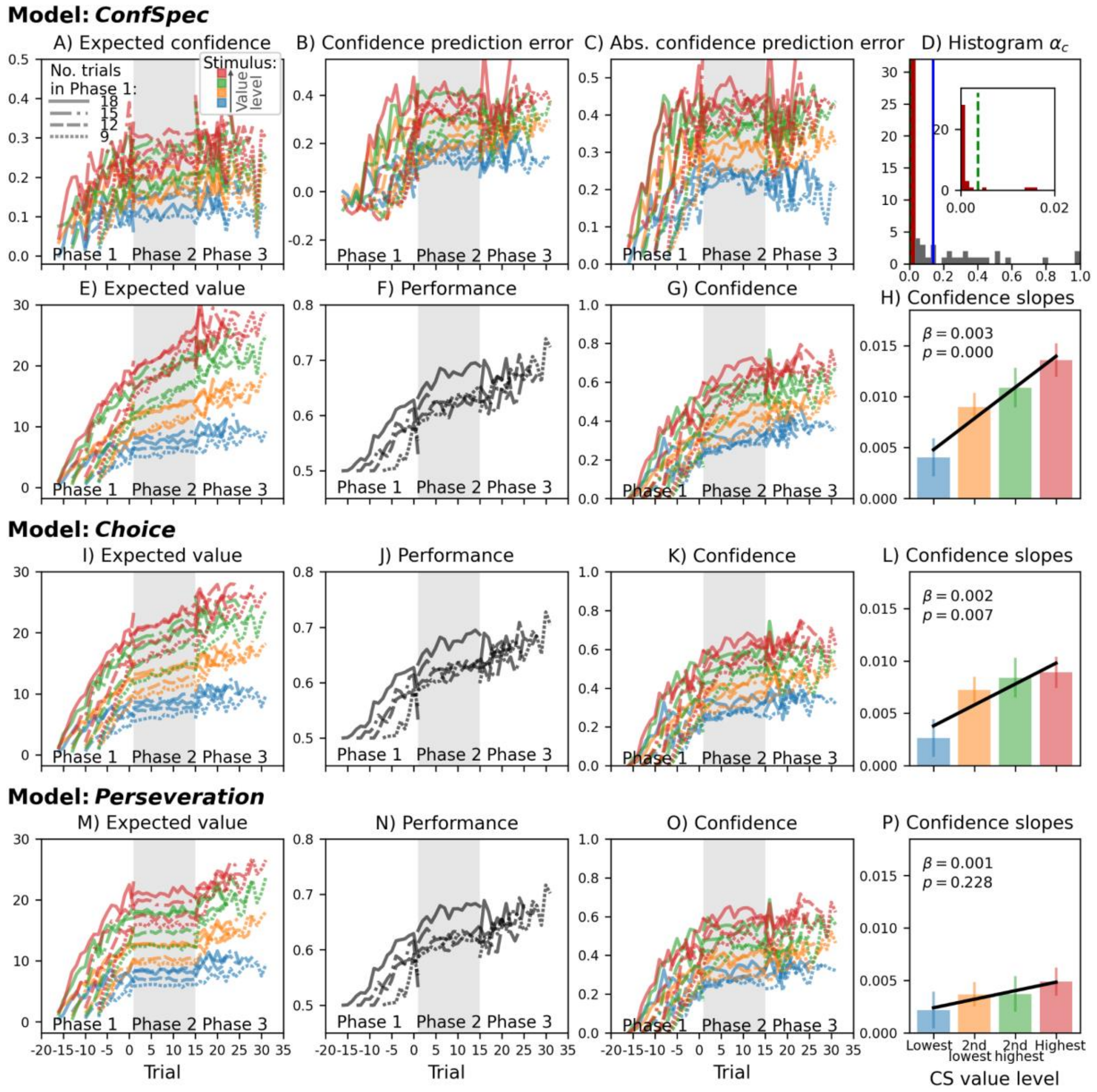

(New) Supplementary Figure S4. Latent variables and behavioral predictions of the models *ConfSpec*, *Choice* and *Perseveration*. See Figure 5 for details.

- The winning model relies on AIC comparison. In Fig 4, is it average AIC across participants? because 4 points of AIC difference may not be much. Have the authors looked at e.g. BIC or model evidence too?

Could the authors perform model recovery, to ensure that their fitting procedure actually can retrieve each of the models separately?

>> The AIC comparison in Figure 4 indeed refers to the average AIC across participants. To clarify the figure content, we now added "Each value represents the average AIC of a model across participants (± SEM)." to the figure description. A delta of 4 may not be a large difference, but it was statistically significant (see paired t-test between the first- and second-best model, p. 15, l. 261 of the revised manuscript). As recommended by the reviewer, we now also computed the Bayesian information criterion for each model fit. The result is included as Supplementary Figure 2 and shown below, for convenience. In brief, also in terms of BIC *ConfUnspec* is the winning model, although the order of the non-winning models is different between BIC and AIC. We mention this supplementary analysis on p. 15, l. 262-264.

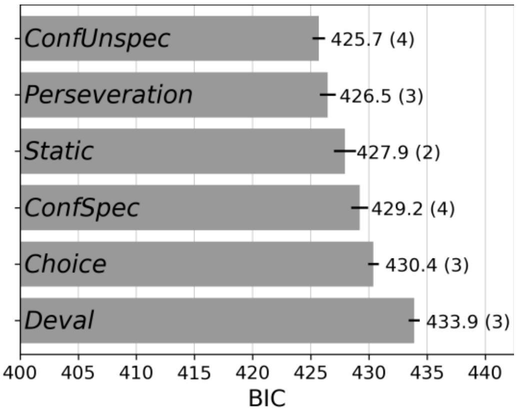

(New) Supplementary Figure 2. Model comparison with the Bayesian information criterion (BIC). Numbers in brackets indicate the number of parameters.

To ensure that each model can be retrieved by our fitting procedure, we now performed a systematic model recovery analysis. To this aim, we simulated data with each of the 5 models of interest, thereby using the parameters obtained from the fit to the behavioral data. Each dataset was then fitted with each of the five models and the Akaike Information Criterion was computed. The resulting confusion matrix is now included as a new Figure 4B (see also below). The matrix demonstrates that we can successfully recover the models *ConfUnspec* (our winning model), *ConfSpec*, *Choice*, *Static* and *Perseveration* (a model suggested by reviewer 2). The only exception is the dataset generated with the *Deval* model, which is best explained by the *Static* model. However, this is explained by the fact that we used the original parameters from the behavioral fit for which the devaluation learning rate parameter α_d (the only difference to the *Static* model) was mostly zero; the *Static* model thus rightly is the more parsimonious model in this case.

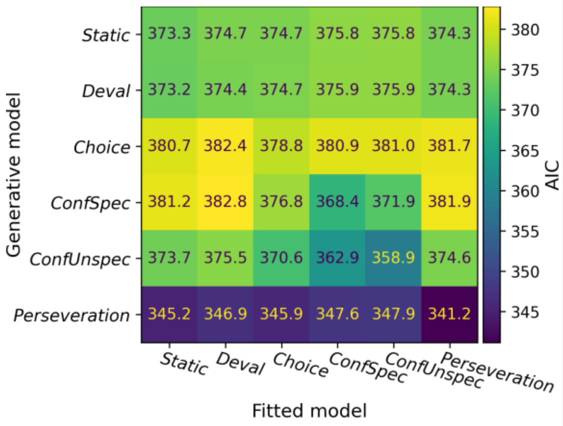

(New) **Figure 4B.** Confusion matrix.

We now refer to this analysis in the Results section (p. 17, l. 278-291).

- Even for validation of the best-fitting model, comparing phase 2 in Fig 2B (participants) and Fig 5B (winning model) seems to reveal different qualitative features: the analyses indicate that confidence increases across phase2, but Fig 2B shows that confidence remains very flat; could the authors explain how we could reconcile this?

>> We agree that the increase in behavioral confidence in the no-feedback phase is relatively subtle – but nevertheless, it is statistically significant (see analysis p. 8, l. 144-145). Unfortunately, the way in which we originally plotted the behavioral confidence time course (a rolling average) masked the confidence increase to a certain extent, which may have induced some cognitive dissonance in relation to the relatively clear statistical effect. See the new Figure 2B for an improved and more veridical presentation. Finally, the basic confidence effect induced by phases without feedback is also supported by the fact that confidence in the last phase is higher in blocks that did include versus did not include a no-feedback phase (p. 8, l. 146-148).

Regarding the mentioned strong(er) increase of expected confidence in the model (Figure 5B), several points should be clarified. First, 'expected confidence' is not the same as 'confidence': confidence only gradually feeds into expected confidence via the model's confidence update rule. Yet, second and as anticipated by the reviewer, the model indeed shows a more pronounced increase of confidence (Figure 5F) compared to the behavioral data (Figure 2B). However, it is important to note that we did not fit confidence ratings in our approach and thus a comparison of absolute values between model-based and behavioral confidence is not readily possible (only a relative comparison, e.g. between value levels). Third, on a more speculative note, it is not unlikely that there are psychological factors that prevent participants from reporting substantially higher confidence in phase 2 (e.g. trying to be consistent with one's prior confidence ratings).

In addition, as described in our response to the previous comment, we now also show that the specific pattern in terms of *how* behavioral confidence increases as a function of the overall item value, is also

predicted by the model. Overall, we thus argue that there is sufficient qualitative correspondence between the metacognitive dynamics of the model and our behavioral data.

It seems important that the authors (i) can better isolate the winning model quantitatively or (ii) can show different qualitative predictions for the behavior of the model as compared to the other models or both (i) and (ii). This would make the claims stronger, or change the implications of the paper quite drastically (in case the winning model no longer wins).

>> In the analyses outlined above, we have aimed at addressing both the qualitative and the quantitative aspects raised by the reviewer. In terms of qualitative predictions, while we could not provide a critical discerning feature between the self-reinforcement models, we could show that all models predict a key qualitative behavioral characteristic concerning the value dependence of a self-reinforced confidence increase. In terms of quantitative analyses, in the updated manuscript we demonstrate successful model recovery (thus also confirming our winning model) and parameter recovery (see our response to Reviewer 2).

General comments

Could the authors comment on the distribution of learning rates for confidence, that they are so low? Is that because of the confidence transfer parameter doing most of the job? Are these two parameters correlated? It would help to get a bit more insight on the mechanism here.

>> Indeed, for quite a few participants the confidence learning rates are relatively small, and upon further reflection, this is an important observation. We now comment on it in the manuscript (p. 18, l. 303-308):

> "[...] that the learning rates for expected confidence (α_c) are relatively small for quite a few participants (Figure 6D), such that expected confidence reflects the increase of behavioral confidence only with a delay. For those participants the expected confidence only slowly increases from its initial value of zero and thus the learning signal (confidence minus expected confidence) is well approximated by confidence itself for a certain setting period."

Of note, the confidence transfer parameter does not need to take a compensatory role for these participants, since a low confidence learning rate does not imply a small learning signal. As described in the Methods section (p. 35, l. 657-659), α_c and γ were largely uncorrelated (winning model: $r = -0.05$, $p = 0.712$).

Regarding the assumption that confidence-related value updates only occur in the absence of external feedback, have the authors tried to extend this mechanism to the feedback case? That is, even if I receive a big reward, I might incorporate the related reward prediction error differently according to whether I was initially sure or unsure about my choice.

>> We agree that it is an intriguing idea that confidence might also modulate the (degree of) learning from feedback-induced reward prediction errors. However, we feel that this is beyond the scope of this paper in which our explicit goal was to investigate the learning dynamics in the absence of external feedback. The fact that we could also test the feedback case is more an accidental side effect since we needed an initial training phase with feedback to set the stage for the no-feedback case. We now added this idea as an outlook in the discussion (p. 28, l. 513-519):

> "An assumption made in the present study is that self-reinforcement is restricted to instances without external reinforcement or cognitive feedback. However, this assumption was not explicitly tested and, at least from a conceptual point of view, the proposed self-reinforcement mechanism in our models could be readily implemented as a modulation of (external) feedback-based model updates, or as a mechanism parallel to those. To test this possibility experimentally, one could introduce an alternative phase 2 that is matched in every respect except the fact that external feedback is provided."

Could the authors comment on the fact that confidence-unspecific model may be appropriate for Phase2 which is limited in time and after which feedback resume, although in real-life situations where lack of feedback is more continuous, would they expect the confidence-unspecific mechanism to be less satisfactory, with a spreading of values across all items? The adaptiveness of this mechanism is commented on a bit in the Discussion, but would deserve more unpacking.

>> Many thanks for raising this relevant question about the ecological validity and scope of the results. As mentioned in the text, we indeed considered it a priori more likely that the learning signal is based on an item-specific confidence baseline. We now comment on this point in the discussion (p. 25, l. 443-449):

> "The evidence for an unspecific maintenance of expected confidence suggests that it is more akin to a tonic state of mind, which is not coupled to simultaneous specific instances of learning. A real-life scenario would be practicing a single task (e.g. a new motor task) over an extended period, such that expected confidence continuously and meaningfully tracks the internally evaluated learning progress. On the other hand, the model predicts that learning without feedback is impaired if multiple parallel tasks are performed that differ in their subjective or objective difficulty."

In the introduction, I was not sure how hypothesis #1 of increase of choice confidence is supported: is it the direct consequence of hypotheses #2 and #3 by which preferences become more marked and therefore choices easier? Or is there another mechanism at play here?

>> The hypothesis regarding an increase of confidence is a consequence of self-reinforcement of choice options and thus indeed a consequence of hypotheses #2 and #3. We had put the confidence effect as #1 since it naturally was the first effect to present in the results, but we now see that the order is a source of confusion in the introduction. We therefore changed the order in the introduction and added a little explainer in brackets for the confidence hypothesis (p. 6, l. 105-108):

> "We therefore hypothesized that the absence of feedback would lead to 1) an augmentation of initial preferences ("the rich get richer and the poor get poorer"), and as a result to 2) an increase of choice consistency and 3) an increase of choice confidence (as preferences become more defined)."

I did not understand what a positive effect of CS repetition number means; in what is this a hallmark of choice consistency? Doesn't it mean that the same CS is selected within the CS pair? Relatedly, I don't understand how Fig 3A is built; why do some consistency values not have a 2nd-3rd choice (orange) bar? Given the randomisation done, should it happen at least a bit?

>> Choice consistency indeed refers to whether the same CS is repeatedly selected within a particular CS pair. With the "positive effect of CS **pair** repetition number" we described the fact that choice consistency increased over the course of phase 2; in other words, choice consistency was higher for the (n+1)th repeated presentation of a CS pair compared to the (n)th presentation (n = CS pair repetition number). We now clarified this with a subordinate clause (p. 9, l. 157-158):

> "[...] , where the repetition number n refers to the *n*th repetition of a CS pair in phase 2."

Please note that the x-axis in Figure 3 indicates consistency probability, where consistency probability is computed as the average frequency of consistent choices in repeated presentations of a CS pair. The histogram illustrates the increase of choice consistency by showing (across subjects) the distribution of consistency probabilities between (i) the first and the second occurrence of a CS pair (in blue), and (ii) between the second and the third occurrence (in orange). It is not expected that each consistency probability occurs for (i) and (ii) (although we believe that this assumption was based on a misunderstanding).

Could the authors unpack their exploratory analysis : what is "value dependency", and why does that show that longer phases without feedback have a "stronger effect of value on rating change"?

>> We agree that our original phrasing ("In an exploratory analysis, we found that the value dependency significantly increased over the course of phase 2[...]") lacked clarity. This analysis referred to our hypothesis that subjective value changes about items in phases without feedback are dependent on the overall value of the item. While, contrary to our hypothesis, we did not find a significant main effect of value, in this exploratory analysis we assessed a potential interaction between the length of phase 2 (i.e. the phase without feedback) and value, which turned out to be significant. In doing so, we made use of the fact that the duration of phase 2 was variable in our experiment (5, 10 or 15 trials). This result suggests that longer phases without feedback might be necessary to produce a measurable value dependency of rating changes.

We now replaced the confusing "over the course of phase 2" with a technically correct description (p. 10-11, l. 177-178):

> "In an exploratory analysis, we found that the value dependency of rating changes **showed a significant positive interaction with the length of phase 2 [...]"**

In terms of the precise model, we refer to Supplementary Table S7. The critical result in this linear mixed regression model analysis is the *value* x *block_ntrials_phase2* interaction effect on the dependent variable *rating_change*.

For choice devaluation (eq7), does it apply only to chosen stimuli, or both chosen and unchosen stimuli?

>> The mechanism of choice devaluation, as any mechanism that we consider, only applies to the chosen stimuli. We now clarify this when we first introduce the value variable v_i ("[..]value v_i of the **chosen stimulus** ***i***"; p. 11, l. 188). In addition, we now also state it explicitly after Equation 7 ("As before, the update rule only affects the chosen stimulus *i*."; p. 13, l. 228-229).

To provide evidence for a similarity between reward-learning and confidence-learning, it seems to me the most straightforward test would be to correlate learning rate reward and learning rate confidence parameters. This correlation is actually provided as a control analysis, but the focus is on correlation between learning rate reward and confidence transfer parameters. Could the authors explain this?

>> Our hypothesis was that observers who more erratically update their subjective values based on external feedback would also do so based on internal feedback. The critical parameter for the internal part is the confidence transfer parameter γ, since this is the parameter that controls the volatility of confidence-based value updates. Note that we could have used the label "learning rate" also for the confidence transfer parameter, as it is also based on a Rescorla-Wagner-type rule for value updates (Eq. 5). Because of this possible source of confusion, we had originally considered using the labels "confidence-based value learning rate" (for the transfer parameter γ) and "confidence-based

confidence learning rate" (for α_c), but decided against it, because the terms could easily be mixed up (and also because of their lengthiness).

The initialisation of expected values and expected confidence are zero, but it seems to me that it would be reasonable that participants expect values to be in the middle, and confidence too, given that after some block they know that they could learn; do these initial values matter a lot of the trajectories of model variables?

>> Here we disagree with the reviewer, because (i) stimuli changed in each block and (ii) participants were informed about this fact. Thus, for the first few choices of each block the initial expected confidence should be zero. Indeed, the average (behavioral) confidence rating for the first encounter of a CS pair was 0.74 on a scale from 0 to 10.

Minor comments

The reward schedule seems clear from the description in Methods, although could the authors comment on a difference in performance across blocks in light of transfer effects? Could the authors confirm that the analyses focus on within-block comparison of the different phases, otherwise this might be problematic for across-block comparisons?

>> For convenience, we below provide a plot of the average performance across blocks. There was no measurable performance transfer between blocks.

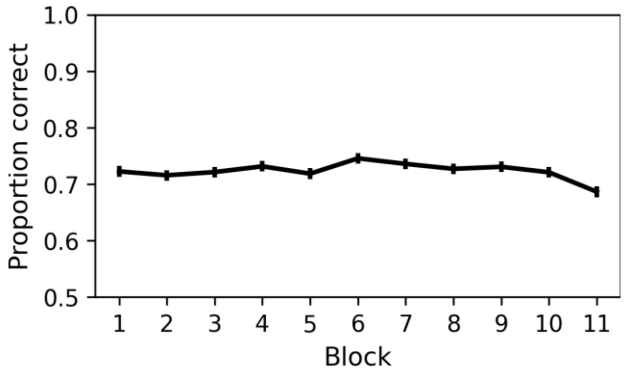

Figure for responses only. Average performance across blocks (error bars = SEM).

We now briefly mention the absence of a transfer effect on p. 32, l. 600-601:

> "To avoid a learning transfer between blocks, different reward schedules were applied (and indeed there was no main effect of block on performance, $p > 0.5$)."

Moreover, we confirm that all analyses are concerned with within-block comparisons.

It would help in the text that the authors clarify what processes are the computational models trying to explain, particularly what aspects of the behavior (seeing Fig2B and 3B)?

>> We took this comment as an opportunity to explain in the modeling section that our computational model indeed is designed to account for (i) value-dependent changes in subjective ratings (Fig. 3B) and increasing choice confidence (Fig. 2B):

> "Overall, the mechanism of self-reinforcement described by Equations 3-5 augments initial preferences (which might have emerged in a phase with feedback) such that initially more preferred options are further positively reinforced and less preferred options are less reinforced or even negatively reinforced. As a consequence, the value landscape becomes more defined and ensuing choices between choice options are made with higher confidence." (p. 12, l. 210-215)

I was unsure about the assumption that dissonance will be stronger for choices that are harder, was it a finding or a hypothesis? I did not understand to what extent the present proposal was consistent or inconsistent with the original Festinger idea. Could the authors unpack their reasoning?

>> To be clear, we did not base our study on the cognitive dissonance framework, but mainly alluded to the conceptual similarities. The notion that "dissonance" is stronger for choices that are harder is from Festinger himself. Indeed, he considers a certain difficulty of choices as a sine qua non for dissonance, as only such choices induce conflict (Festinger, 1957, p.77). In general, we view our findings as inconsistent with Festinger's cognitive dissonance theory and unpack this in the discussion (p. 27-28, l. 499-507):

> "According to Festinger, subjective values are increased for chosen options (and decreased for unchosen options) as a form of post-hoc rationalization, to reduce the dissonance that would arise otherwise when reflecting on the positive attributes of an unchosen option. The larger the dissonance, the stronger the expected preference changes. Since the dissonance will be stronger for choices that are subjectively perceived as harder, those choices should be associated with a lower level of choice confidence. Thus, Festinger's theory predicts that higher choice confidence leads to higher preference changes for the chosen option, whereas our proposed model predicts the opposite (note however, that our model does not consider changes for the unchosen option)."

In Fig 4 what are error bars? Are bars and error bars average and S.E.M. or S.D.?

>> Yes, the bars represent the average and the error bars s.e.m. We now added this information to the figure description.

Sometimes 'variables' is used where 'parameters' is meant, I believe

>> Many thanks for pointing us towards this inconsistency. In the context of our discussion of the Beta distribution we have referred to the shape values α and β of the Beta distribution both as parameters and as variables. The source of confusion here is due to the fact that while α and β are generally the *parameters* of the Beta distribution, they are here used as *variables* (which are updated from trial to trial) in this specific context. However, we have since deleted the corresponding paragraph from the discussion due to space constraints (we have extended other parts of the discussion at the recommendation of both reviewers).

Why does the upper bound of softmax inf too?

>> If we understand correctly, the question is why we chose an upper bound of 2 for the softmax parameter. Due to the scaling of the values, softmax parameters are expected to be in the range of 0.1 and the upper bound of 2 is thus a very liberal constraint. We chose this upper bound as a form of regularization, to tame the parameter values of participants who are not fit well by the model. Indeed, there was a single participant whose psychometric function was not fit well and who was affected by this upper bound (cf. Figure 6B).

Reviewer #2: In this manuscript, Ptasczynski and colleagues investigate behavioral and computational aspects of learning without feedback revealed by choices and confidence judgements. They used a simple instrumental-learning task which includes phases with and without external feedback and in which participants (n= 64) reported their confidence in addition to choices. The authors report signatures of self-reinforcement in phases without feedback, reflected in an increase of subjective confidence and choice consistency. They propose to account for these findings with a confidence-based learning models, which they compare with other potential models.

I found the manuscript generally well written, and appreciate the author care in providing an appropriate sample size (N = 64 – quite large for lab experiments) as well as a comprehensive analytical strategy.

Yet, and although I anticipate that the general topic, research question and approach would trigger some interest in the field, I feel that the current version of this manuscript falls short of providing a convincing demonstration of the effects and a compelling interpretation of the findings. I also have more conceptual interrogations about the general research question. I detail these concerns below.

>> We thank the reviewer for an immensely instructive and in-depth review. We also appreciate the general skepticism. We have now performed new analyses to catch up with current computational modeling practices and have also addressed the reviewer's other concerns as thoroughly as possible. We hope to convince the reviewer that the work provides a strong conceptual contribution and the appropriate methodological rigor, even if some of the behavioral effects are not as strong (which we couldn't change in this revision).

Main concerns:

- First, I am having a hard time trying to figure out what exactly is (conceptually) this proposed self-reinforcement without feedback, in the context of this task. Is it an (normative) adaptation to some specific kind of ecological situations encountered in ecological settings ? Is this a bias ? Currently, it feels a bit like the research question is a bit of an ad-hoc effect, with an ad-hoc model. In other words: why should we expect confidence to rise in the absence of feedback ? Is there a limit to this increase ? The authors seem to frame this as a natural mechanism but I can imagine a lot of situations where the proposed mechanism would lead to terrible decision-making…

>> We understand the reviewer's reservation about the ecological rationale of self-reinforcement without feedback and apologize that this was not well contextualized in the previous version of the manuscript.

First, the reviewer's question about whether self-reinforcement is adaptive or could constitute a maladaptive bias is spot-on. As outlined in the introduction, the idea of self-reinforcement comes from

an entirely different domain (perceptual learning). In this domain, we (Guggenmos et al., 2016; Guggenmos and Sterzer, 2017) and others (Herzog and Fahle, 1998; Seitz and Watanabe, 2005; Daniel and Pollmann, 2012; Diaz et al., 2017) have shown that a self-reinforcement mechanism is plausible from a computational point of view and fits well with experimental data. Early on, this self-reinforcement mechanism has been proposed to rely on a diffuse global internal reward signal (Herzog and Fahle, 1998; Seitz and Watanabe, 2005) and indeed, two subsequent studies have provided evidence for an involvement of the (likely dopaminergic) mesolimbic reward system in generating such internal feedback signals (Daniel and Pollmann, 2012; Guggenmos et al., 2016).

This earlier research is the starting point for the present study, in which our goal was to probe whether such signatures of self-reinforcement signals in the absence of external feedback can also be detected in value-based decision making. We chose value-based decision making specifically, because it is a form of learning in which dopaminergic learning signals play a well-established and crucial role. Our research question is thus far from ad-hoc, but is well motivated from our previous research; likewise the model, which shares core computational components (e.g. confidence prediction errors) with previous models.

Now, we completely agree that prima facie self-reinforcement in value-based decision making does not constitute adaptive behavior, as it mainly strengthens pre-existing biases. Although we discuss a potential adaptive benefit of such self-reinforcement in the face of memory leakage / retrieval-induced forgetting (p. 23, l. 392-395), we are completely open to the possibility that the proposed mechanism, while useful in some domains, could be maladaptive in the context of value-based decision making – not least because it is a domain prone to a number of cognitive biases (cf. our discussion of the cognitive dissonance phenomenon, p. 27-28, l. 499-507).

We realize that in the previous version we have not sufficiently unpacked this line of reasoning, which we hope clarifies the derivation of our research question. We now updated the introduction and the discussion accordingly:

> “We hypothesized that, if confidence in actions indeed takes the form of a diffuse internal reward signal, it may also affect the subjective values of these actions, similar to instances of external reinforcement. What could be the benefit of such a self-reinforcement mechanism in the context of value-based decision making? Although we prefer to be agnostic at this point about whether such a mechanism would be adaptive, self-reinforcement could strengthen previously learned preferences to make them more robust in the face of decision noise and potential memory leakage. On the other hand it is possible that such a mechanism is a general automatic concomitant of learning and decision making without external feedback that, while beneficial in some domains, may not be useful or even maladaptive in others, including value-based decision making.” (p. 4-5, l. 74-83)

> “[...] while it may seem irrational when choice options are transformed into a simplified categorical scheme of either good or bad options, such a scheme may actually be more robust

towards mnemonic deterioration. Indeed, in the absence of a memory loss mechanism, the winning model *ConfUnspec*, like the two other self-reinforcing models *ConfSpec* and *Choice*, show that self-reinforcement can even lead to a (potentially compensatory) increase in performance as values become more defined (cf. Figure 5A). Such a scenario could explain the behavioral effects of stable type 1 performance with a parallel increase of confidence and choice consistency." (p. 23-24, l. 402-410)

Finally we'd like to note that the prediction error mechanism in our model prevents confidence from increasing ad infinitum, even in the case of the model with unspecific expected confidence. As expected, values continue to spread and thus confidence and expected confidence increase, the learning signal (confidence minus expected confidence) will eventually seize to zero.

- I am also a bit unconvinced by the mere behavioral effects. Although the GLMM indicate a significant effect of trials on confidence in phase 2, the Figure 2B pictures an extremely small trend. Actually, it almost seems that confidence in the first trial of phase 3 is actually lower than (or at least no higher than) the confidence in the first trial of phase 2 – this would indicate that the apparent increase in confidence in the phase 2 is more a psychological effect of the absence of feedback than a self-reinforcement mechanism. Likewise, the effect on choice consistency are not very convincing: why limiting the analyses to trials 1-3 of phase 2 (Figure 3A)?

>> We agree that the increase in behavioral confidence in the no-feedback phase is not very strong. However, regarding the mere visual appearance of the effect, we noted that the original confidence plot has masked the confidence increase to a certain degree through the use of a (incorrectly applied) rolling average. The corrected plot in Figure 2B (without a rolling average) is more in line with the relatively clear statistical result (p. 8, l. 144-145). While a certain momentary drop in confidence is still visible, as soon as feedback is switched back on in phase 3, the confidence in the first trial of phase 3 (average 0.43) is still higher than in the first trial of phase 2 (average 0.40). Moreover, the general confidence effect induced through phase 2 is supported by the fact that confidence in the last phase is higher in blocks that did include versus did not include this phase (p. 8, l. 146-148).

Finally, for this revision we now assessed a more nuanced metacognitive prediction of self-reinforcing models which is that the increase in confidence should be dependent on the overall value level. We show via model simulation that all three self-reinforcing models (*ConfUnspec*, *ConfSpec*, *Choice*) predict this dependence (Figure 5G and Supplementary Figures 4H, L; see also our longer response to the first comment of the other reviewer). Crucially, we observe the same effect in our behavioral data (Figure 2C). This analysis provides independent evidence that the behavioral dynamics of confidence are not random.

Regarding the final point, we would like to clarify that our analysis of choice consistency is not limited to trials 1-3. The repetition numbers 1-3 refer to the first, second and third occurrence of a specific CS

pair (consistency is computed as the frequency of a repeated choice within the same CS pair). We thus analyse the entire phase 2, but given its fairly limited duration, CS pairs do not occur more than three times. However, our previous description was a bit confusing and we have adapted the corresponding sentence to make this more clear ("[..], where the repetition number n refers to the nth repetition of a CS pair in phase 2; p. 9, l. 157-158).

One could actually compute a trial-by trial measure of choice repetition, and evaluate (and actually illustrate with a time-series/learning-curve figure) if/how choice repetition actually increases over the 20 trials of phase 2 – which should be the case under the authors' hypothesis. This would correspond to the analysis reported in Table S5, which currently show a borderline effect (p = 0.032), but with some anomalies in the table (reported coefficient estimates are not included in the reported confidence intervals). Finally, and as transparently acknowledged by the authors (lines 151-160), there is no significantly detectable effect on value rating. So, overall, it seems that the model-free experimental evidence in favor of the self-reinforcement without feedback is at best tenuous. Of course, the data is the data (and I am not encouraging the authors to p-hack some new pattern of results here) – but in the absence of e.g. a strong replication, I will remain skeptic about the reality (and interpretation) of the behavioral effect.

>> The constraint of the limited number of repetitions of each choice pair explains the type of illustration chosen in Figure 3A. The number of different choice pairs (10) is too high to allow for a meaningful learning curve across trials. For this reason, the IV of interest in Table S5 is not trial number, but choice pair repetition number (*trial_pair_repeat_nr*). As outlined in our response to the reviewer's last minor comment, the confidence intervals in all regression tables with a binary DV (which includes Table S5) were erroneous.

We agree that the lack of an effect of objective stimulus value on rating change is not in favor of our overall interpretation of a self-reinforcing mechanism. One reasonable explanation for the observed differences in rating and model behavior might come from the fact that participant's value estimate was blurred by our introduction of noise or uncertainty into the reward display (*N* of coins was drawn from 1SD distribution around its mean), while the computational models are more sensitive to small numerical differences. This perceptual uncertainty might have led to the case, where small objective value differences were harder to identify for participants as compared to the computational model since a delta of 3 or 4 coins is difficult to perceive, especially across trials. Note that, in addition to that, coins were randomly spread across the reward display panel avoiding that participants were able to "count" the absolute *N* of coins and introducing another source of difficulty.

Overall, while we acknowledge that our behavioral evidence could be more convincing, we believe that the larger pattern of behavioral data provides sufficient justification for our approach: (i) an increase of confidence despite the absence of new information, (ii) a highly specific interaction of value and

confidence increase, and (iii) an increase of choice consistency. Also note our comment on the subjective value result to the next but one reviewer concern. Moreover, the fact remains that a self-reinforcement model outperforms a number of control models, now including an additional choice perseveration model (see below). Nevertheless, to better acknowledge the behavioral effects sizes, we now explicitly mention it as a limitation in the Discussion (p. 28, l. 520-527):

> "A limitation of the present study is that most behavioral effects are not very strong, including an absent main effect for the predicted change of subjective values, which was significant only for the longest duration of phase 2. This suggests that self-reinforcement effects in the absence of external feedback are relatively subtle, or, more unfavourably for the present study, a false positive. Either way, it is clear that investigating the choice and confidence dynamics in the absence of external feedback calls for large sample sizes. Moreover, our results suggest that the emergence of self-reinforcement effects at the level of conscious report might require no-feedback phases of sufficient length."

- This allows me to transition to my second line of concerns – about the (model-based) interpretation of the effects. I am very puzzled by the winning, candidate model put forward by the authors. First, there are several aspects of the model rationale that I find questionable: e.g. the fact that there is confidence "transfer" only during phase 2 (one can imagine that this also happens when feedback is present), or that the choice probability (i.e. the softmax output) rather than the actual choice is used to determine the CS to which the confidence-value transfer is applied (lines 552-553).

The author justifies it (lines 554-555) by explaining that using the actual choice would not make sense for the model, but it feels to me that this is not a good justification: one should adjust the model to the theory, rather than adjusting the theory to the constraints of the model.

>> We agree that it is an intriguing idea that confidence might also modulate the (degree of) learning from feedback-induced reward prediction errors. However, our explicit goal of this study was to investigate the learning dynamics in the absence of external feedback. The fact that we could also test the feedback case is more an accidental side effect since we needed an initial training phase with feedback to set the stage for the no-feedback case. We now mention this as an avenue for future research in the discussion (p. 28, l. 513-519):

> "An assumption made in the present study is that self-reinforcement is restricted to instances without external reinforcement or cognitive feedback. However, this assumption was not explicitly tested and, at least from a conceptual point of view, the proposed self-reinforcement mechanism in our models could be readily implemented as a modulation of (external) feedback-based model updates, or as a mechanism parallel to those. To test this possibility experimentally, one could introduce an alternative phase 2 that is matched in every respect except the fact that external feedback is provided."

Regarding the reviewer's second point, we must clarify that using simulated rather than behavioral choices is inevitable for the type of model we have proposed. The reason is stated in the manuscript (p. 34, l. 640-641): reinforcing choice options that were actually chosen by the participant would provide the model with privileged information about the true preferences of participants (a choice is an indication of a true preference). Using the actual choices would also be circular, since, at the same time, we fit the model to the participants' choices. Indeed, we had initially used the actual choices and the (unrealistic) high level of model evidence that resulted from this pointed us to the issue. Thus, we do not adjust the theory to the constraints of the model, but we run the model in a technically valid and unbiased procedure. Strictly speaking, this procedure is to our own detriment, as it introduces some degree of noise into the process (based on the fact that a certain proportion of simulated choices will be different from the actual choices).

In connection to my general conceptual point, I also feel that the proposed model does not fully account for the observe behavioral patterns – this is maybe due to the fact that the authors only fit the model, and do not explore its generative performance (see (Palminteri et al., 2017)). For instance, if the model contributes to an evolution of option's expected values (Figure 5A) shouldn't it predict de facto a detectable increase in performance during phase 2 ?

- Given that the behavior actually falsifies the idea that the value (value ratings) change during phase 2, I have the feeling that a better (and more legitimate) model to explain the increase in choice consistency would be one leveraging a choice perseveration bias in the absence of feedback (possibly with the perseveration or choice temperature that is modulated by confidence) – see e.g. (Correa et al., 2018; Katahira, 2018; Rutledge et al., 2009).

>> While we agree that our behavioral effects are generally not very strong, we do not think that the subjective value ratings, which are certainly subject to all kinds of biases and noise sources, constitute a falsification for the idea of self-reinforcement. Although not a strong argument, we also point out that the value ratings numerically show the predicted effect and the effect becomes significant when the phase without feedback is long enough (cf. Supplementary Figure 1).

However, we quite like the idea of the reviewer of testing a choice perseveration model, as formalized in the provided references. The promising advantage of this model is that it indeed predicts constant performance across phase 2 (since − as opposed to self-reinforcing models − values are unaffected). We now implemented such a model (referred to as *Perseveration*) and included it in our list of models:

> "Finally, we tested a model in which choices likewise become more consistent in the absence of external feedback, but in which the actual values are unchanged. This is accomplished by means of a choice perseveration bias parameter η (55,56), which captures tendencies to perseverate (positive values) or alternate (negative values). The parameter η of this *Perseveration* model

affects choice probabilities and is described in the Methods section on 'Model parameters and model fitting'." (p. 13, l. 230-235)

"In case of the *Perseveration* model, the choice probability in the absence of external feedback contains a perseveration bias as follows (55,56):

$$p_{right} = \frac{1}{1 + e^{-\beta\,[(\bar{v}_{right} - \bar{v}_{left}) + \eta\,(C^{prev}_{right} - C^{prev}_{left}\ )]}} \qquad (10)$$

where $C^{prev}_{left/right} = 1$ if the CS corresponding to the CS on the left/right side was chosen in the previous encounter of the CS pair (0 otherwise). An individual with a positive/negative parameter η would have a bias towards repeating/alternating the previous response." (p. 34, l. 642-646)

Note that we implemented the model as stated in the provided references. A modulation by confidence of this perseveration bias seems counterintuitive since the computation of confidence comes after the choice.

We confirm the constant performance effect of the *Perseveration* model in our exploration of the generative behavior, which we now included at the recommendation of both reviewers (Figure 5 and Supplementary Figure 4N). However, the model does not predict an increase of confidence or a modulation of the confidence increase by value (Supplementary Figures 4O, P), thus partly falsifying the model.

We find that the model performs better than the self-reinforced *Choice* model, but still inferior to the two self-reinforced confidence models *ConfUnspec* and *ConfSpec* (Figure 4A). Of note, we find that all participants show some 'choice stickiness', as indicated by the fact that the perseveration parameter η is positive for all participants (η=0.73±0.05, cf. Table 2).

- Finally, the model-based analysis does not really comply with current, state of the art modeling practices, and should include parameter recovery & model identification analyses (Wilson and Collins, 2019), as well as model simulations/falsifications (Palminteri et al., 2017).

>> We agree that our initial manuscript lacked key analyses that are now state-of-the-art in computational modeling. As outlined partly in our other responses, we now performed both a systematic model identification and parameter recovery analysis, as shown by the results in the two new Figures 4B and Supplementary Figure S3 (pasted below, for convenience). In brief, we find successful model identification and parameter recovery (see also our longer response to the first comment of reviewer 1).

We report on the new model identification and parameter recovery analyses in the model comparison section as follows (p. 17, l. 278-291):

> "To ensure that our fitting procedure was able to retrieve each model, we performed a systematic model recovery analysis. To this end, we simulated data with each of the five models of interest, thereby using the parameters obtained from the fit to the behavioral data. Each dataset was then fitted with each of the five models and the Akaike Information Criterion was computed. The resulting confusion matrix (see Figure 4B) demonstrates that we can successfully recover the models *ConfUnspec* (our winning model), *ConfSpec*, *Choice*, *Static and Perseveration*. The only exception is the dataset generated through the *Deval* model, which is best explained by the *Static* model. However, this is explained by the fact that we used the original parameters from the behavioral fit for which the devaluation learning parameter α_d (the only difference to the *Static* model) was mostly zero; the *Static* model thus rightly is the more parsimonious model in this case. Overall, this analysis demonstrates successful model recovery. In addition, we performed a systematic parameter recovery analysis for the winning model *ConfUnspec*, which confirmed that our model fitting procedure infers generative parameters in an unbiased manner (Supplementary figure S3)."

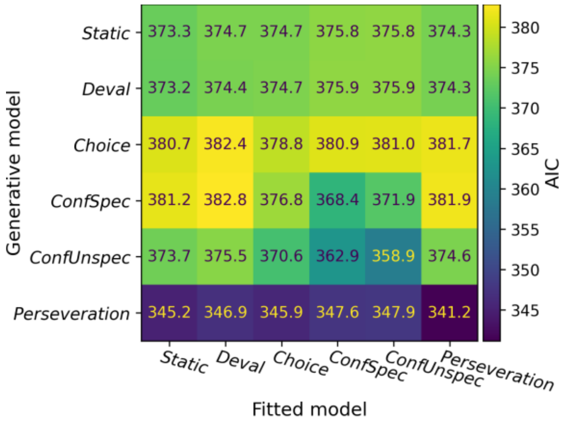

(New) Figure 4B. Confusion matrix.

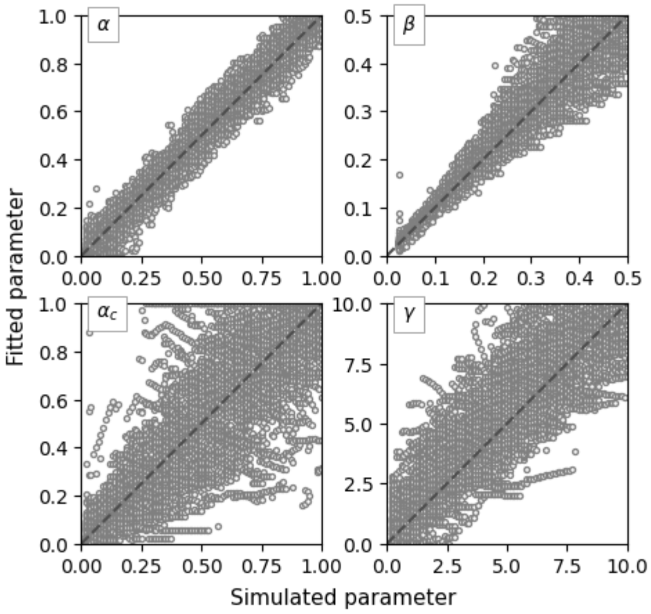

(New) Supplementary Figure S3. **Parameter recovery.** Simulated versus fitted parameters for the winning model ConfUnspec.

Other concerns

- The use of AIC as the model-comparison criterion seems a arbitrary. This should be backed up, at the very least, by a model identification analysis (Wilson and Collins, 2019), or dropped for more principled model-comparison metrics. Likewise, the whole model-comparison exercise seems to consist in a random effect analysis of AIC (t-tests), which is not very standard nor principled. Some more principled solutions exists – see e.g. (Daunizeau et al., 2014).

>> We'd like to point out that the paper recommended by both reviewers on several occasions (Wilson and Collins, 2019) advocates the use of either AIC or Bayesian information criterion (BIC) for model comparison. Between AIC and BIC, to our knowledge, it is not settled which (if any) is more suited for cognitive computational models, and an entire literature exists debating the assumptions and applicable domains of both measures (e.g. Aho et al., 2014). Following the recommendation of reviewer 1, in the new version of the manuscript we now also report a model comparison using the BIC metric. This analysis confirms the *ConfUnspec* model as the winning model. While the use of a t-test may not be standard for the comparison of AIC/BIC scores, we think that it is to be preferred over a simple numerical comparison of AIC/BIC scores as done in many papers. However, we agree that a model identification analysis is important and warranted for our approach and its lack was a shortcoming of the previous version of the manuscript, which we now rectified (see our response above).

- The initial values of expected values and confidence are set to 0 (544-545). Given that the task features only cues that have a positive expected value, shouldn't v0 (which correspond to participants prior

expectations) also be strictly positive (e.g. an average between the worst and best outcome) ? Likewise, it seems that initializing expected confidence at 0 does not really correspond to participants behavior/initial beliefs (Figure 2B).

>> We see the reviewer's point, but would argue that our choice of initializing expected confidence and expected value with zero is pragmatic and justifiable. While the participants indeed do not start with exactly zero in terms of their reported confidence, in our view it is sufficiently close to zero to justify a parsimonious model without a dedicated initial confidence parameter (the average confidence is 0.74 on a scale from 0-10 for the first encounter of a CS pair). Initializing expected *values* with zero is a standard procedure since the idea of instrumental conditioning is that CS which have not yet received any reinforcement, are not loaded with subjective value. The type of expectation that the reviewer suggests, which could result from participants building an internal model of the task structure, would be rather cognitive in nature. If taken seriously, such a process would require a more complicated model that captures the build-up of initial values across the experiment, since a v0>0 is hard to justify for the first block. But more importantly, it requires the additional assumption that this type of cognitive expectation is comparable to subjective values resulting from a reinforcement process. Given that our focus here is on the phase without feedback, we prefer to not make relatively strong assumptions about the initial phase in which participants receive feedback.

- There are some issues with the GLMM results reported in Tables S3 and S5: the reported coefficients are not included in their respective CI.

>> Thank you for noticing this error. These were the models that we computed in R. What happened is that while transferring the data to our Python pipeline, we mistook the odds ratio confidence intervals for the coefficient confidence intervals. We have corrected this mistake.

- The authors report (lines 134-135) "By contrast, confidence increased across phase 2 (5.75 ± 0.04 [s.e.m.]; LMM: z = 3.12, p = 0.002; Figure 2B and Supplementary Table S4)". I do not understand what the 5.75 ± 0.04 stands for.

>> The values referred to the average (unnormalized) confidence ± s.e.m. in phase 2. This information was misplaced, as this sentence refers to an *increase* of confidence, hence the confusion. We removed it.

References:

Aho K, Derryberry D, Peterson T (2014) Model selection for ecologists: the worldviews of AIC and BIC. Ecology 95:631–636.
Daniel R, Pollmann S (2012) Striatal activations signal prediction errors on confidence in the absence of external feedback. NeuroImage 59:3457–3467.
Diaz JA, Queirazza F, Philiastides MG (2017) Perceptual learning alters post-sensory processing in human decision-making. Nat Hum Behav 1:0035.
Guggenmos M, Sterzer P (2017) A confidence-based reinforcement learning model for perceptual learning. BioRxiv.
Guggenmos M, Wilbertz G, Hebart MN, Sterzer P (2016) Mesolimbic confidence signals guide perceptual learning in the absence of external feedback. eLife 5:1–19.
Herzog MH, Fahle M (1998) Modeling perceptual learning: difficulties and how they can be overcome. Biol Cybern 78:107–117.
Seitz AR, Watanabe T (2005) A unified model for perceptual learning. Trends Cogn Sci 9:329–334.
