# Supplementary material for: The value of confidence: Confidence prediction errors drive value-based learning in the absence of external feedback
Source: PLoS Comput Biol. 2022 Oct 3;18(10):e1010580. doi: 10.1371/journal.pcbi.1010580 (PMC9560614; doi:10.1371/journal.pcbi.1010580)
Supplement: Attachment — Submitted filename: Ptasczynski_et_al_Response_to_reviewers2_PDF.pdf [file pcbi.1010580.s010.pdf]

Reviewer #1: I have now carefully read the responses to my comments for the study of Ptasczynski and colleagues, and I found that the manuscript has substantially improved.

My enthusiasm is a bit mitigated by the fact that the three main models do not present critically distinctive behavioral predictions, and, less importantly, they do not explain all aspects of behavioral data; although I understand that the study was not designed to arbitrate between these three models, that the data are what they are - and the text and claims of the paper are commensurate to the strength of the findings. Here are a few remaining points.

>> We thank the reviewer for their constructive comments which once again clearly improved the manuscript.

The authors discuss the adaptiveness of their identified self-reinforcement mechanism in terms of being potentially protective against memory leakage. In my understanding, this claim is mitigated by the three self-reinforcing model being consistent on most behavioral patterns. At the authors note, it is difficult to provide a functional interpretation for the adaptiveness of ConfUnspec because it might depend on the context. I appreciate that the authors' model comparison indicates that BIC and AIC favour the ConfUnspec model, but still, in the absence of strong qualitative differences in behavior, it is difficult to appreciate where this differences in AIC/BIC come from. Instead, could the authors adapt and focus their discussion (lines 429-449) on the implication of the three self-reinforcement mechanisms compatible with the behavioral data, rather than of ConfUnspec specifically?

>> As the reviewer notes, we indeed suspect that the adaptive benefit of a self-reinforcing mechanism would be to protect preferences against memory leakage, in particular those that we are ourselves confident in (*ConfSpec*/*ConfUnspec* model) or that are more frequently chosen (*Choice* model). We didn't quite understand why the similarity of our self-reinforcing models would mitigate this interpretation. However, we do agree (and perhaps this was the point) that we cannot argue that this potential benefit is a specific and discriminative feature of the *ConfUnspec* model.

In response to reviewer 2, we have now performed a more systematic analysis of model generative performance, across different parameter regimes. While this analysis yielded a few interesting insights about differential qualitative behaviors of the three models, they likewise do not provide a basis for strong conclusions about the specific mechanism of self-reinforcement. For this reason, we followed the recommendation of the reviewer and now cut the (longish) part of the Discussion about the specific properties of the *ConfUnspec* model.

In addition, we discuss the ambiguity about the specific self-reinforcement mechanism (lines 471-495):

> "However, the similarity of posterior predictive fits between the self-reinforcing models *ConfUnspec*, *ConfSpec* and *Choice* (Figure 5 and Supplementary Figure S4A-L) gives reason to be cautious about a specific mechanistic interpretation of the behavioral effects. While only the posterior predictive fits of the three self-reinforcing models showed the *Confidence x value effect*, there is no 'smoking gun' that discriminates between these models. Our analysis of model generative performance provided only partial resolution in that the *Confidence x value effect* was found not to be a natural property of the *Choice* model and is produced only under specific parameter settings for the *ConfSpec* model. Thus, the *Confidence x value effect* arises most naturally in the *ConfUnspec* model. The fact that this effect is more robust across different parameters for the *ConfUnspec* model means that these parameters have more flexibility which could be a potential explanation for the superior model fit.
>
> The present work thus provides evidence that value-based learning in the absence of external feedback is shaped by some form of self-reinforcement, but the *specific* proposed mechanism of the *ConfUnspec* model is mainly supported by our quantitative model comparison and not by a clear falsification of the other self-reinforcement models. Only the *Choice* model falls off to a certain degree as it does not generally produce a *Confidence x value effect* and thus doesn't support our proposed adaptive mechanism of self-reinforcement, i.e. protecting against memory leakage in phases where subjective preferences are not refreshed by external feedback. Combined with the fact that the *Choice* model did not perform well in the quantitative model comparison, ranking even behind the *Perseveration* model, we suggest that a confidence-based rather than a mere choice-based self-reinforcement mechanism is likely. Disambiguating between the two confidence models might necessitate an experimental paradigm that is tailored to the differences between these models, for instance by introducing conditions that manipulate the degree to which the unspecific prediction error references of the *ConfUnspec* model are problematic for learning (e.g., 'roving' conditions; 37)."

However, note that we kept part of the text within the lines referenced by the reviewer (lines 429-449) in which we expressed our surprise about the fact that the *ConfUnspec* model was *quantitatively* superior to the *ConfSpec* model (lines 460-470):

> "In the logic of the best-fitting computational model (*ConfUnspec*), subjective values of chosen CS are reinforced if, and only if, choice confidence is higher than expected on the basis of previous confidence experiences, i.e. in the case of positive confidence prediction errors. By contrast, chosen CS are devalued if confidence prediction errors are negative. It is noteworthy that the *ConfUnspec* model, i.e. a model with an unspecific reference (*expected confidence*) to which momentary confidence levels are compared, outperformed a model in which expected confidence was CS-specific. We considered this unlikely, a priori, since an unspecific reference deprives the confidence prediction error from its natural convergence property: an unspecific reference maintains the average confidence level across all CS so that prediction errors, in principle, can be persistently positive (for CS judged to be of relatively high value) or negative (for CS judged to be of relatively low value)."

We believe that this aspect deserves highlighting even if the posterior predictive fits of the different models were similar.

In response to Reviewer 2, I note that the parameter recovery is encouraging but incomplete. It is not sufficient to show that each of the 4 parameters can be captured, but that each parameter captures itself * and not the other * or * better than the other * parameters. From the correlations between fitted and simulated parameters in Fig S3, the authors can compute a form of 4x4 matrix, akin to the model recovery, but this time for parameter recovery. In other words, we currently see the correlations (simulated parameter i, fitted parameter i), and we need to see the correlations for all pairs (simulated parameter i, fitted parameter j).

>> We thank the reviewer for this helpful explanation, which clarified the request. We now computed the full parameter correlation matrix. To this end, we systematically varied each parameter between reasonable minimum and maximum values and computed the Pearson correlation between all generative and all fitted parameters. To test whether parameter recovery was robust against different settings of the respective other parameters, we additionally performed this analysis for a coarse parameter grid consisting of five different values for each of the four parameters. We decided to visualise parameter recovery in dependence of β and γ specifically, as those most sensitively affected the quality of parameter recovery. See below, for the new Supplementary Figure S7.

We find that parameter recovery generally works well, supporting our previous analysis. Please note that the depicted correlation matrices represent an overall average across different settings for the other two parameters α_r and α_c including settings that make model recovery harder. In the text, we describe this updated analysis as follows (lines 709-721):

> "To assess the quality of parameter recovery for the winning model *ConfUnspec*, we generated datasets for which we systematically varied each model parameter between sensible lower and upper bounds (250 equidistant values). We fitted the model to each dataset and computed the full correlation matrix of fitted and generative parameters. To make the analysis robust against the specific settings of the respective other parameters, we performed this process for each node of the coarse parameter grid of size 5^N described above.
>
> Supplementary Figure S7 shows that parameter recovery works well across various parameter regimes. Two edge cases deserve mention. First, we found that too low values for the confidence transfer parameter γ impair the ability to recover the confidence learning rate α_c (if confidence has little effect on value estimates, the learning rate carries little weight either). Second, if choices become increasingly random (small values of β), recovery is likewise impaired to a certain degree. Nevertheless, for typical parameter values in our empirical data parameter recovery is sufficiently precise."

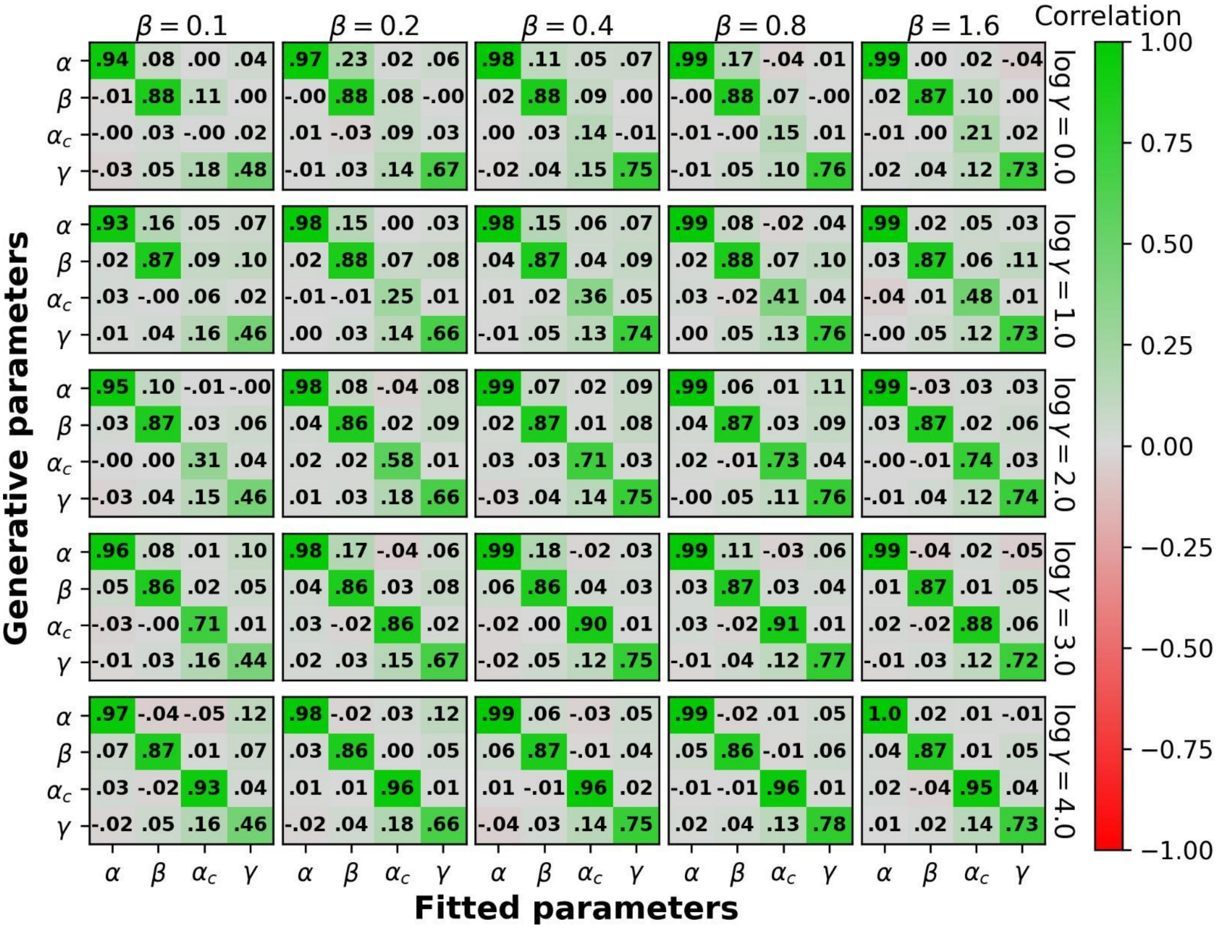

Supplementary Figure S7: Parameter recovery. Pearson correlation matrices between generative parameters and fitted parameters in dependence of different settings for β and γ.

Minor comments

In their Discussion, could the authors explicitly clarify the unique specificities of the present learning mechanism as compared to the learning mechanism proposed in Guggenmos et al 2016 eLife?

We now explicate the difference between the models in lines 213-220:

> "The proposed model differs from the original perceptual learning model (12) in terms of how a diffuse confidence prediction error signal takes effect: in the perceptual learning model, confidence prediction errors shaped the weights of a simple sensory processing network, requiring a Hebbian learning component in equation (5) to ensure differential effects on signal and noise weights (known as a three-factor learning rule; 31). Since the present value-based decision task does not involve processing of a perceptually ambiguous stimulus, the model architecture is simpler and requires only the standard one-factor prediction error learning rule of equation (5)."

It would be useful to match the panels between e.g., Fig 5 and S4A, 2B and 5F to facilitate comparisons.

>> We thank the reviewer for this suggestion. We now matched the order of panels between Figures 2, 5 and S3 (previously S4).

I find the notion of “more erratically update” ambiguous: could the authors replace with e.g. “with more variability”?

>> We agree and replaced “more erratically” with the neutral descriptor “more variable” (see line 328).

Reviewer #2: Ptasczynski revised their manuscript about confidence-driven learning in the absence of external feedback, to address the issues raised about their original submission by other reviewers and myself. First, I would like to thank the author for their attempt to address thoroughly and constructively the said issues: obviously, and lot of time and effort has been invested, and I find the revised manuscript much improved. Yet, in my view, some specific points of concern are still not satisfactorily addressed. I will try to re-state the analyses that I feel are still missing in the paper (or currently, mis-specified) and that would be needed to be added or corrected for me to recommend the manuscript for publication. All relates to the quality-control of the modelling.

>> We thank the reviewer for their thorough and patient review, which we took as an opportunity to extend and improve our modelling practice.

1. Model generative performance. As far as I understand, the authors still not provide the simulation exercise required my myself and other reviewers, pertaining to model generative performance or model falsification (Palminteri et al., 2017). In response to these concerns the authors have produced graphs of the model latent variables / posterior predictive fits / fitted behavior. Although those are important per se, they do not address the question of model generative performance: those should rely on pure simulated behavior – and basically check that the model can generate the behavioral pattern of interest. What is the difference ? Simply, checking model simulated behavior rather than model fitted behavior. Why is this important ? Especially in RL, models tend to overfit behavior, due to the auto-correlation in the choice patterns. To understand this point, (Palminteri et al., 2017) makes a compelling point that e.g. Win-Stay Lose-Shift or choice-repetition models can fit learning behavior very well, but generally fail to produce the desired process of interest (e.g. reversal learning). Here because the proposed model really is a mechanism for the observed pattern of behavior, one need to be convinced that it can produce it, via pure simulations.

>> We thank the reviewer for clarifying the desired analysis. We now realise the importance of assessing model generative performance more generally, i.e. independent of the specific fitted parameters. As outlined in the following, these analyses indeed help assess under which settings each model produces the observed behavioral effects.

We describe our new analysis as follows in the Methods section (lines 723-739):

“To better understand the qualitative behavior of the four best fitting models (*ConfUnspec*, *ConfSpec*, *Choice*, *Perseveration*) we simulated choice and confidence data across different parameter settings. Specifically, we systematically varied parameters that influence the behavioral dynamics in phase 2, i.e. α_c (range 0-1 in steps of 0.5) and log γ (range 0-4 in steps of 1) for the *ConfUnspec* and *ConfSpec* model, λ (range 0-10 in 8 exponential steps) for the *Choice* model and η (range −1.5-1.5 in steps of 0.5) for the *Perseveration* model. Since we found that the effects of interest in phase 2 are not sensitive to the precise settings of α_r and β (which shape behavior in phases with feedback), we fixed both parameters at 0.2, close to the average in the behavioral data and close to typical values in this context. For each model and parameter combination, 250 datasets were simulated.

Based on the simulated data, we computed four effects of interest: 1) *Performance effect*, defined as the average slope of proportion correct responses across the trials of phase 2; 2) *Confidence effect*, defined as the average slope of confidence across the trials of phase 2; 3) *Confidence x value effect*, defined as the slope for the interaction of CS-specific confidence slopes and corresponding CS values; 4) *Consistency effect*, defined as the increase of the proportion of consistent choices between the first and second occurrence of a choice pair versus the second and third occurrence of a choice pair.”

These new analyses are summarised in the new Supplementary Figure S4:

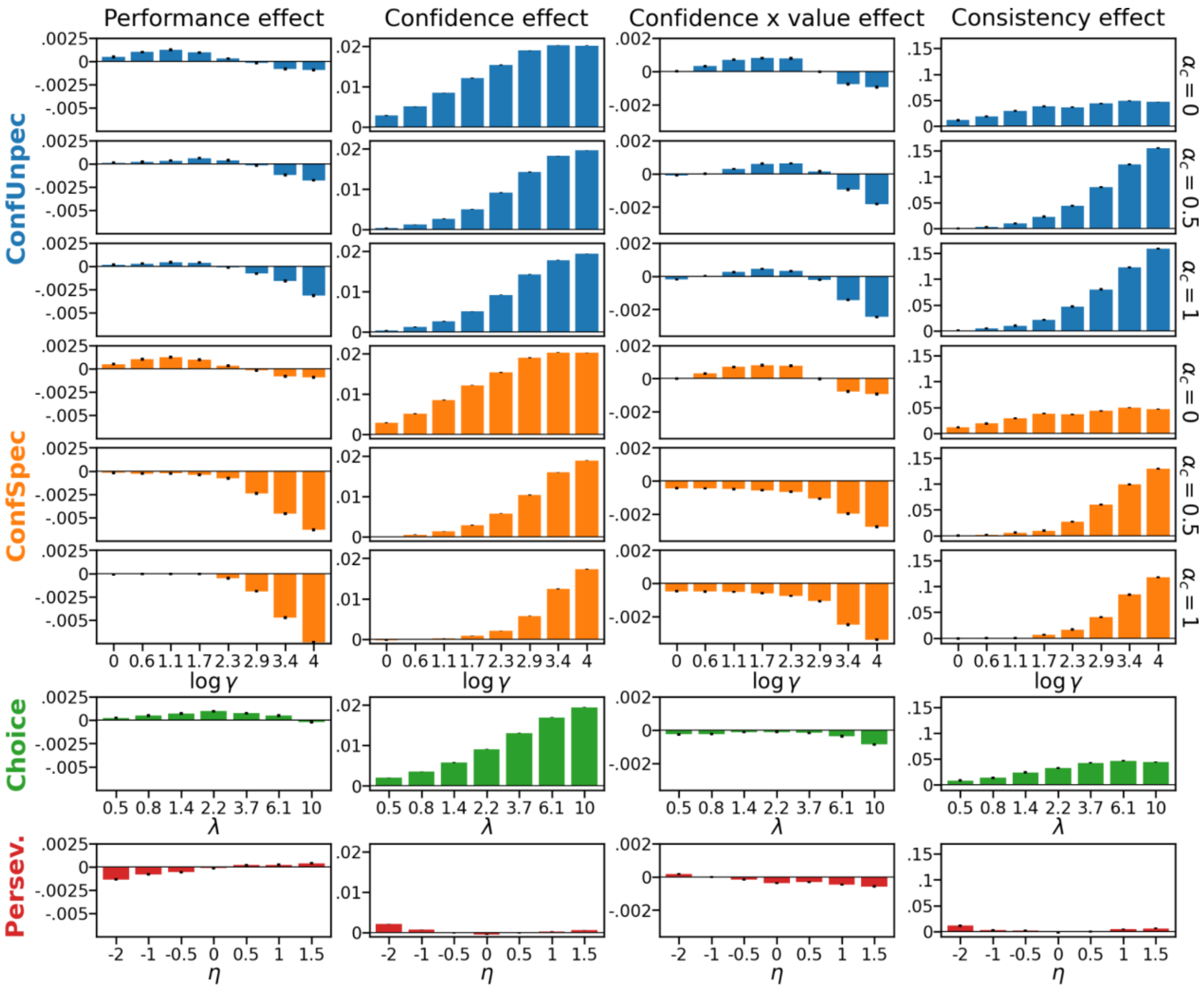

Supplementary Figure S4: Generative model performance for four key effects in dependence of different parameter settings. Model generative performance was assessed for the four best-performing models (*ConfUnspec*, *ConfSpec*, *Choice*, *Perseveration*). *Performance effect*: performance slope in phase 2. *Confidence effect*: confidence slope in phase 2. *Confidence x value effect*: slope for the interaction of the *Confidence effect* and value (cf. Figure 2C). *Consistency effect*: increase in choice consistency between the first and second occurrence of a choice pair versus the second and the third occurrence (cf. Figure 3A). Error bars depict the standard error of the mean across 250 simulated datasets.

We describe this new analysis as follows in the Results section (lines 347-378):

“To clarify whether the behavioral predictions of the models naturally arise from the models or are caused by overfitting, we performed a second analysis in which we more generally assessed model generative performance in dependence of relevant parameter settings (Supplementary Figure S4). As for the behavioural analyses, we assessed changes of confidence and performance in phase 2 (referred to as *Confidence effect* and *Performance effect*), the interaction of confidence slopes and value (*Confidence x value effect*) and changes of choice consistency in phase 2 (*Consistency effect*).

We found that two of the behaviorally observed effects are robustly produced by all self-reinforcing models (i.e. *ConfUnspec*, *ConfSpec*, *Choice*) across parameter regimes: a positive *Confidence effect*, i.e. an increase of confidence across phase 2, and a positive *Consistency effect*, corresponding to an increase of choice consistency. By contrast, the *Confidence x value effect*, i.e. higher confidence slopes for more valuable CS, emerges as a general effect only for the confidence-based models *ConfUnspec* and *ConfSpec*. In addition, the *ConfSpec* model exhibits this effect only for small values of α_c, whereas in the *ConfUnspec* model the effect arises robustly across different settings of α_c. Coincidentally, as noted above, we found that the fitted parameters for α_c were lower (and more frequently close to zero) in the *ConfSpec* model compared to the *ConfUnspec* model. While not allowing for strong conclusions, this observation indicates that the empirically observed *Confidence x value effect* arises most naturally for the *ConfUnspec* model and can only be achieved by special parameter combinations for the *ConfSpec* model. This may be a potential explanation for the superior model fit of *ConfUnspec*.

Compared to the *Confidence effect*, the positive *Performance effect* (which was absent in the empirical data) only emerges under specific parameter combinations for the *ConfSpec* and *ConfUnspec* model. Specifically, the *Performance effect* arises precisely for parameter combinations that also yield a *Confidence x value effect*. This makes sense: when observers become more confident in more valuable CS this should go hand in hand with an increase in performance. Importantly, the *Performance effect* is minuscule with slopes in the order of at most 0.001, which corresponds to a performance increase of 1% across 10 trials. This could explain why no performance effect was observed in the behavioral data, even if behavior was shaped by a confidence-based reinforcement mechanism. Finally, note that the *Perseveration* model does not show any of the discussed effects to a meaningful degree within the relevant parameter ranges.”

Note that we adopted the term ‘posterior predictive fits’ suggested by the reviewer to describe the original analyses in which behavioral model predictions were based on fitted parameters (e.g. lines 288-289). This provides a clear terminological differentiation with respect to the new analyses of model generative performance.

2. As for the falsification (which is again, currently problematically based on model fitting rather than model simulation), the authors currently show that all models predict the same confidence slope as a function of CS value. Can differential patterns be observed as a function of the other task parameters e.g. No trials in phase 1 ? Or interactions between these factors ? I feel that model falsification is a very desirable property, especially in a modelling paper that propose new mechanisms.

>> As a first point we would like to restate that our results clearly falsify the *Static* and the *Devaluation* models, in the sense that naturally neither of these models can produce the observed behavioral effects. The first model predicts no changes in phases without feedback and the second model predicts devaluation of CS that are not ‘refreshed’ by external feedback. We hope that this point is clear without a comprehensive model simulation.

Nevertheless, we understand that the reviewer is interested in whether a simulation could falsify one or more of the self-reinforcing models (or perhaps the *Perseveration* model), thereby qualitatively discriminating between these models. Since we don’t see how one of the task parameters could deliver this purpose (and we certainly did not have an a priori hypothesis about this), we would like to restrict this assessment to the key behavioral effects of interest and the associated simulations presented in response to the previous comment. As this new simulation of model generative performance showed that the *Choice* model does not generally produce the *Confidence x value effect*, and also given the *Choice* model’s inferior ranking in the quantitative model comparison, we reason that a confidence-based self-reinforcement mechanism is more likely than a mere choice-based mechanism. On the other hand, the present work does not (and we think cannot) falsify between the two confidence-based learning models.

In line with a recommendation of reviewer 1, we now discuss this more cautiously (lines 482-495):

> “The present work thus provides evidence that value-based learning in the absence of external feedback is shaped by some form of self-reinforcement, but the *specific* proposed mechanism of the *ConfUnspec* model is mainly supported by our quantitative model comparison and not by a clear falsification of the other self-reinforcement models. Only the *Choice* model falls off to a certain degree as it does not generally produce a *Confidence x value effect* and thus also not our proposed adaptive mechanism of self-reinforcement, i.e. protecting against memory leakage in phases where subjective preferences are not refreshed by external feedback. Combined with the fact that the *Choice* model did not perform well in the quantitative model comparison, ranking even behind the *Perseveration* model, we suggest that a confidence-based rather than a mere choice-based self-reinforcement mechanism is likely. Disambiguating between the two confidence models might necessitate an experimental paradigm that is tailored to the differences between these models, for instance by introducing conditions that manipulate the degree to which the unspecific prediction error references of the *ConfUnspec* model are problematic for learning (e.g., ‘roving’ conditions; 37).”

3. Model identification. Currently, I find the model identification analysis unsatisfactory. First, the metric is average AIC, which is hard to interpret, and can be driven by outliers : I suggest that the authors run several iterations of the modelling exercise, and report the probability of a model being identified as the best through their “preferred” model comparison exercise (p(fit|gen)). This probability is indispensable to infer what the model-comparison obtained from the actual data actually means. Second, it seems that the perseveration model wins all model comparison in the simulations. Actually, to deal with this case, I recommend that the author compute the reverse probability p(gen|fit), which would help the interpretation of the model comparison exercise – see e.g. Extended Data Fig. 5: in (Ciranka et al., 2022) for a similar approach/rationale.

>> We thank the reviewer for these helpful suggestions. We now performed several iterations across a comprehensive parameter grid to test model recovery. We agree that computing recovery probabilities is more robust and more informative and hence now use this new measure for quantification. Please find the new Supplementary Figure S5 below:

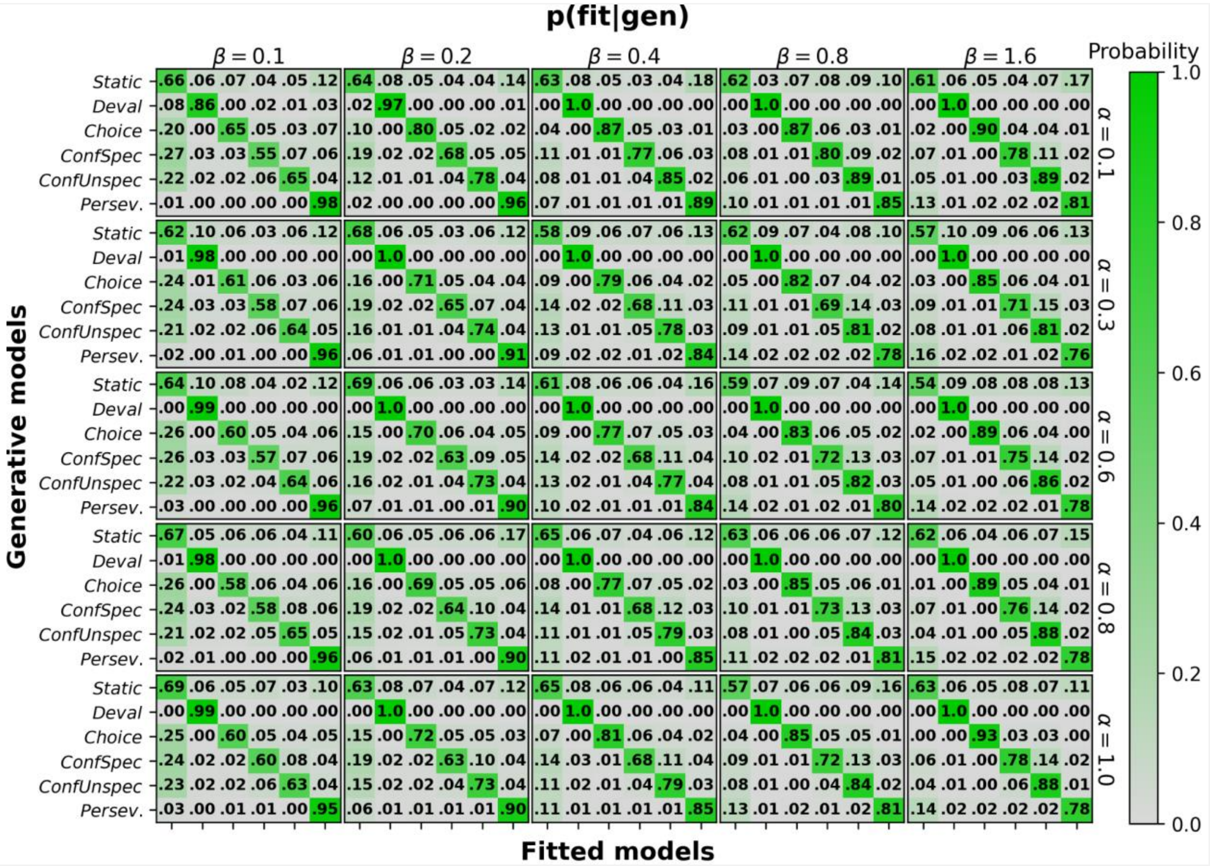

Supplementary Figure S5. Model recovery (1): probability that a generative model *gen* is best fitted by a test model *fit*. Rows represent generative models and each column within a row indicates the probability that a dataset was best fitted by a particular model. Note that the order of models is the same along both axes, but labels were omitted on the x-axis due to space constraints.

Moreover, we also see much value in the second analysis suggested by the reviewer, i.e. computing the reverse probability that datasets best fitted by a particular model were indeed generated by that model − p(gen|fit). We included this analysis as Supplementary Figure S6:

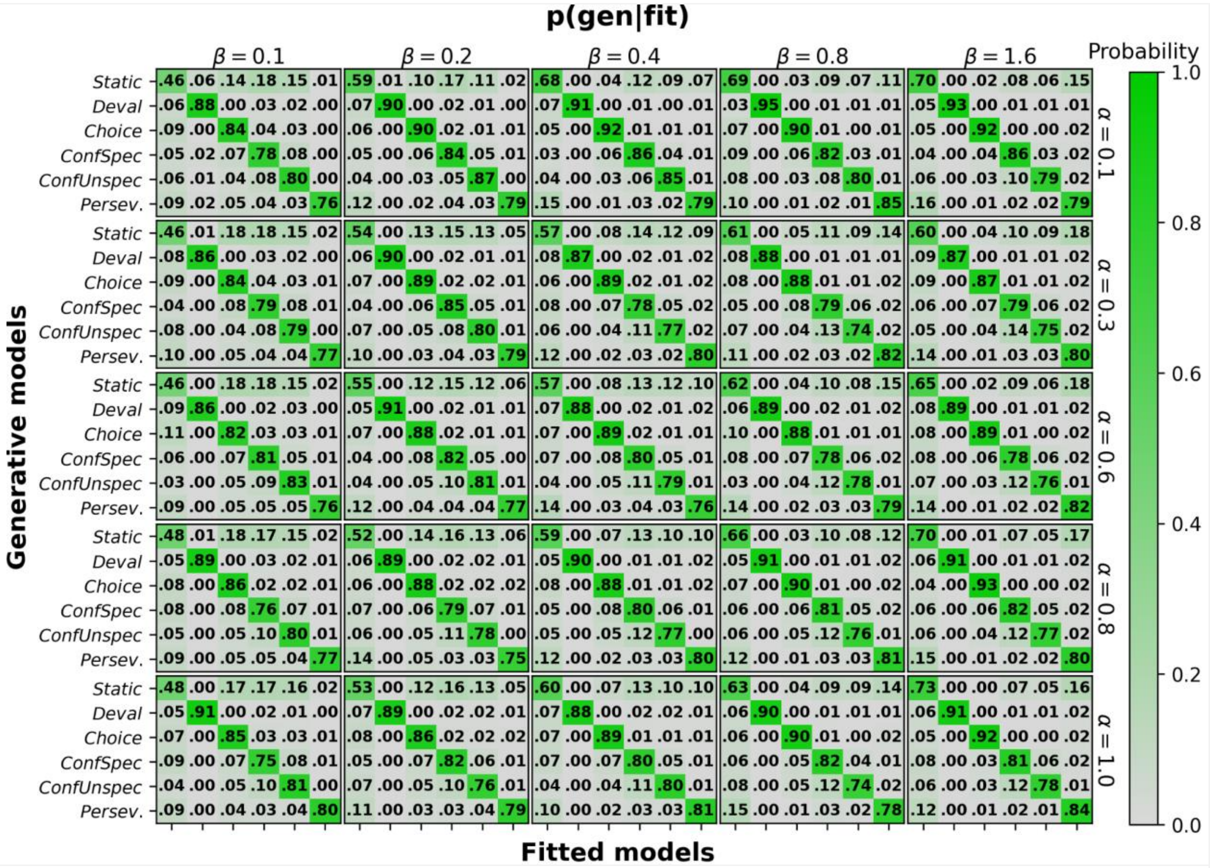

Supplementary Figure S6. Model recovery (2): probability that a dataset best fitted by model *fit* was generated by model *gen*. Rows represent the datasets in which the given model was best-fitting and each column within a row indicates the probability that the datasets were generated by a particular model. Note that the order of models is the same along both axes, but labels were omitted on the x-axis due to space constraints.

We describe and interpret both analyses in the Methods section as follows (lines 679-708):

“To ensure that all models are identifiable with sufficient precision, we performed a model recovery analysis. For each model we simulated choice and confidence data in dependence of different settings for each parameter (5^N different parameter configurations, where N is the number of parameters and 5 corresponds the number of different values for each parameter between reasonable minima and maxima). We generated 250 datasets for each parameter combination. Model recovery was quantified by the probability that datasets generated with a given model X were best fitted by a model Y, p(fit=X|gen=Y), as well as the reverse probability that datasets best fitted by a given model Y were generated by a model X, p(gen=X|fit=Y). For p(gen=X|fit=Y), we made sure that the base rate is identical across models. We computed the probabilities separately for different values of α_r and β, the two parameters that were common to all models.

As shown in Supplementary Figure S5, we found that datasets generated with a given model are also most likely fitted by this model. More precisely, p(fit|gen) is generally much higher when the generative and fitting model are identical relative to when they are different. Perhaps even more importantly, the reverse probability matrix, p(gen|fit), likewise demonstrates good model identifiability (Supplementary Figure S6). That is, datasets that are best fitted by a given model are, in all likelihood, also generated by this model. This latter analysis is an important prerequisite for any conclusion about underlying mechanisms in our empirical data that derive from the superior model fit of the *ConfUnspec* model.

Notably, the model that performs worst in terms of model identifiability is the *Static* model, both in terms of p(fit|gen) and p(gen|fit). In quite a few instances, datasets generated by the *Static* model are confused with one of the other models (except the *Deval* model). This shows that due to random variation and limited trial numbers (which matched the empirical experiment), the choice dynamics are sometimes better described by more complex models; conversely, the *Static* model is sometimes the best-fitting model although the data is generated by more complex models (because the dynamics generated by the complex models are not distinct enough to compensate for the complexity punishment). Importantly, instances in which data is best fitted by our main models of interest (i.e. the self-reinforcing models *ConfUnspec*, *ConfSpec* and *Choice*) correspond most frequently to datasets that are also generated by these models.”

4. Parameter recovery. The parameter recovery analyses currently does not give a fair representation of the ability of the modelling framework to correctly estimate parameters, because parameters are varied/estimated independently. This completely misses the fact that when fitting the data, multiple parameters can jointly compete to explain the same share of variance. For this reason, the parameter recovery should be ran by varying and fitting all parameters simultaneously.

>> We thank the reviewer for clarifying this important point. We now computed the full parameter recovery matrix to also show a potential covariance structure. Moreover, to assess the robustness of parameter recovery across different settings of each respective 'other' parameter, we additionally performed this analysis for each permutation of a coarse parameter grid. This method underlying this revised parameter recovery is explained as follows in manuscript (lines 709-714):

> "To assess the quality of parameter recovery for the winning model *ConfUnspec*, we generated datasets for which we systematically varied each model parameter between sensible lower and upper bounds (250 equidistant values). We fitted the model to each dataset and computed the full correlation matrix between generative and fitted parameters. To make the analysis robust against the specific settings of the respective other parameters, we performed this process for each node of the coarse parameter grid of size 5^N described above."

Note that the final 'described above' refers to the model identification analysis, for which we exercised the same parameter grid.

See below for the result of this analysis:

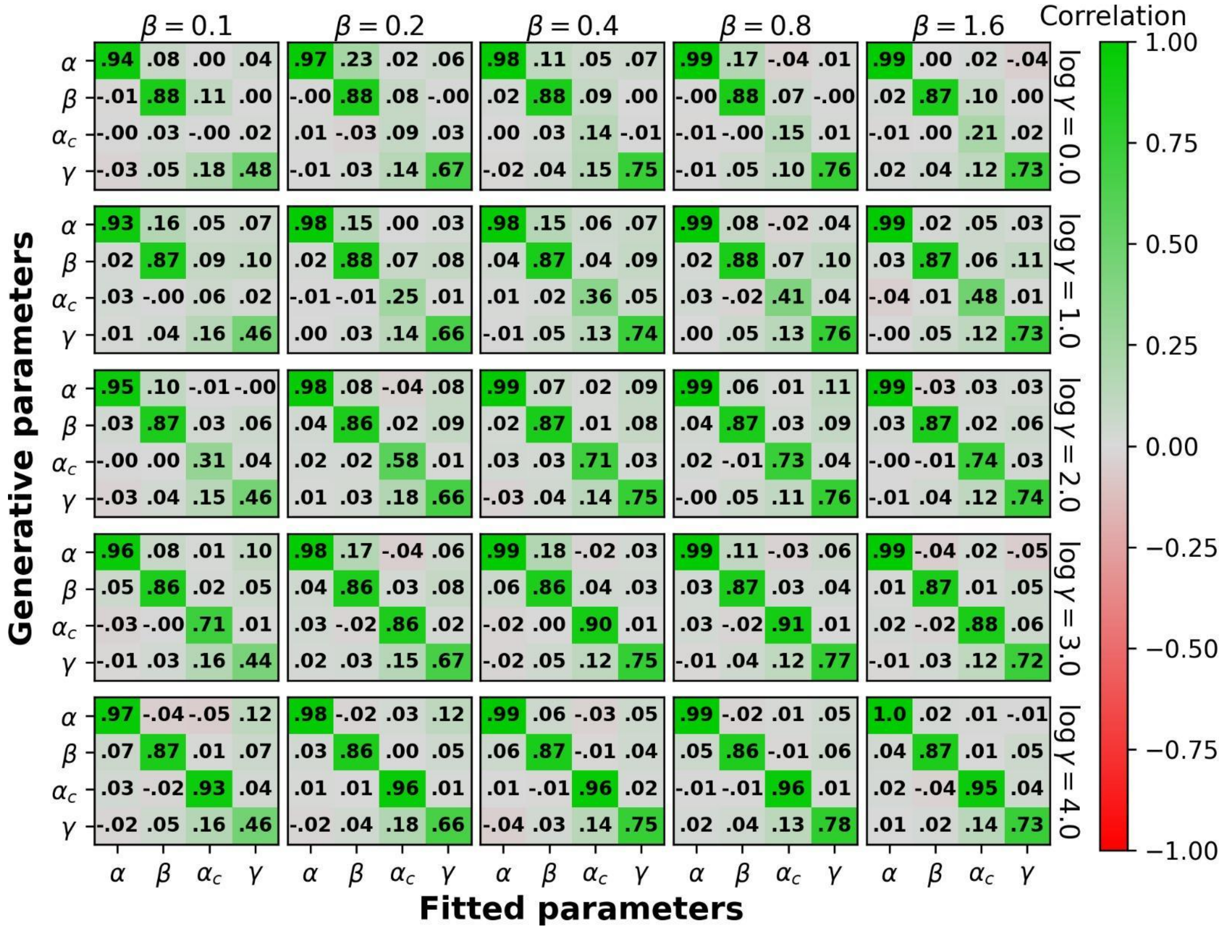

Supplementary Figure S7: Parameter recovery. Pearson correlation matrices between generative parameters and fitted parameters in dependence of different settings for β and γ.

In the text, we interpret these results as follows (lines 715-721):

> "Supplementary Figure S7 shows that parameter recovery works well across various parameter regimes. Two edge cases deserve mention. First, we found that too low values for the confidence transfer parameter γ impair the ability to recover the confidence learning rate α_c (if confidence has little effect on value estimates, the learning rate carries little weight either). Second, if choices become increasingly random (small values of β), recovery is likewise impaired to a certain degree. Nevertheless, for typical parameter values in our empirical data parameter recovery is sufficiently precise."
