# Supplementary material for: The value of confidence: Confidence prediction errors drive value-based learning in the absence of external feedback
Source: PLoS Comput Biol. 2022 Oct 3;18(10):e1010580. doi: 10.1371/journal.pcbi.1010580 (PMC9560614; doi:10.1371/journal.pcbi.1010580)
Supplement: Attachment — Submitted filename: Ptasczynski_etal_Response_to_reviewers3_PDF.pdf [file pcbi.1010580.s011.pdf]

Reviewer #1: The authors have now provided additional analyses regarding the remaining points raised by other reviewers and myself about model validity, which has significantly improved the manuscript.

- Model recovery: I found the new Figure S4 hard to parse; we do not know what behavioral effects of participants are reproduced vs. not, it would be useful to indicate / report more precisely how human data compares.

>> We thank the reviewer for this suggestion. We would like to point out that we discuss quite detailed how human data compares to the simulated one in lines 347–378. However, we agree that it is useful to provide the behavioral effects as a direct reference in Supplementary Figure S4. The updated Figure S4 shown below now indicates the respective behavioral effects as dashed lines (see further explanation in the Figure legend).

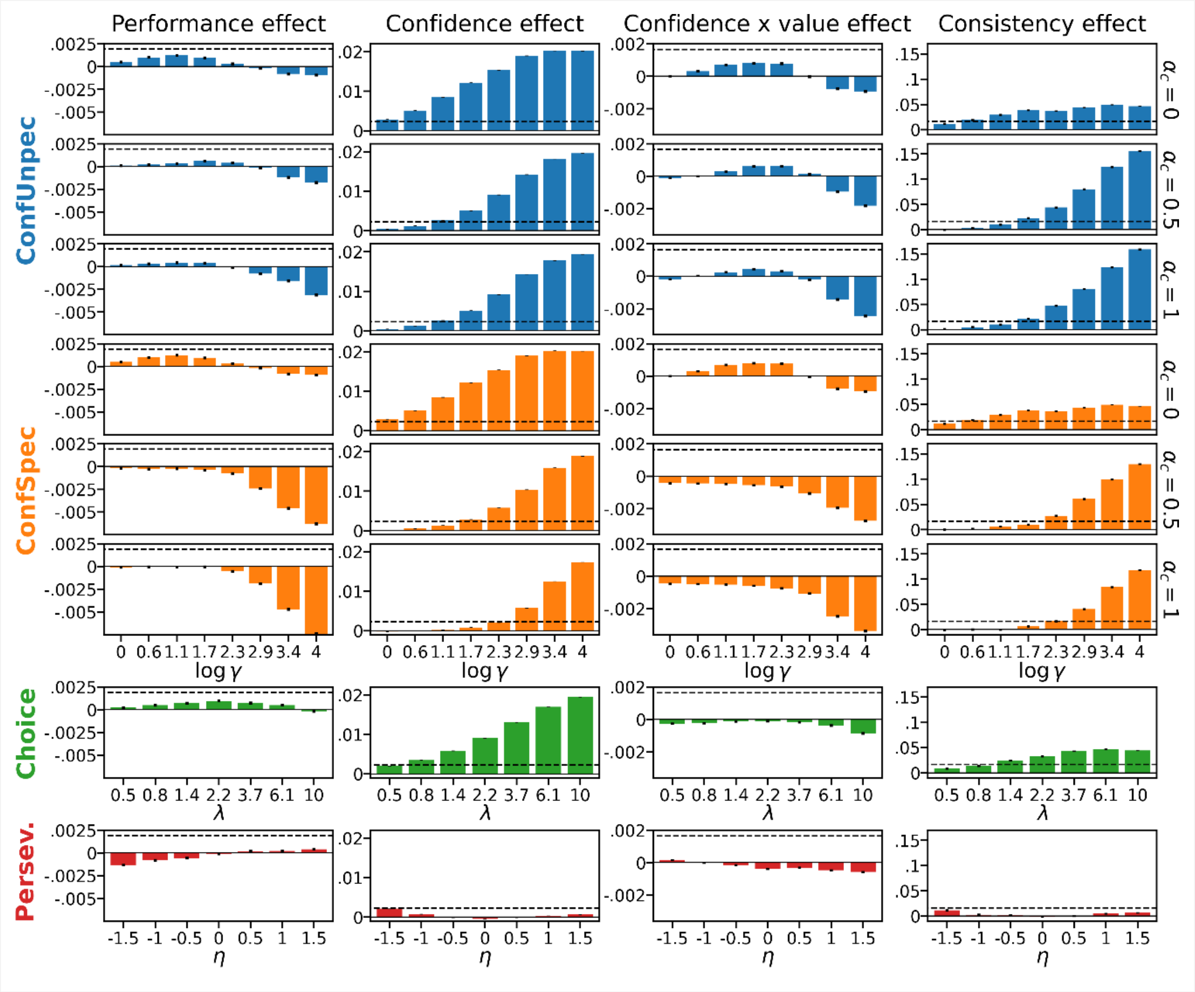

Supplementary Figure S4. Generative model performance for four key effects in dependence of different parameter settings. Model generative performance was assessed for the four best-performing models (*ConfUnspec*, *ConfSpec*, *Choice*, *Perseveration*). Model-based effects are shown as bar graphs, behavioral effects as dashed lines. *Performance effect*: model-based and behavioral graphs depict linear performance slopes in phase 2. *Confidence effect*: model-based and behavioral graphs depict linear confidence slopes in phase 2. *Confidence x value effect*: model-based and behavioral graphs depict slopes for the interaction of the Confidence effect and bandit value (cf. Figure 2C). *Consistency effect*: model-based and behavioral graphs depict the increase in choice consistency between the first and second occurrence of a choice pair versus the second and the third occurrence (cf. Figure 3A). Error bars for model-based effects indicate standard errors of the mean across 250 simulated subjects.

- If I understood correctly, model simulations are not done from best-fitting parameters? It does not have to be independent from the fitted model parameters: quite the contrary, we want to know: within the regime of the best-fitting parameters, whether model simulations based on those parameters are able to reproduce the behavioral patterns of interest. But because the authors have swept across the whole landscape for selecting their parameter values, the relevant regimes are normally included.

>> Indeed, as suggested by the previous reviews, the idea of this simulation was to explore the models' behavior more generally, e.g., to exclude the possibility that the good fit of the winning model *ConfUnspec* is a rather unrepresentative local minimum in the generative performance landscape. However, as the reviewer states, the best-fitting parameters are included in the range of simulated parameters. Please also note that simulations explicitly using the best-fitting parameters are still shown in Figure S3.

- Falsification is not possible between the main candidate mechanisms, as acknowledged by the authors. As in my previous review, my enthusiasm remains mitigated by the fact that the main confidence-based models do not present critically distinctive behavioral predictions, and do not explain all aspects of behavioral data. However, I appreciate that the study was not designed to arbitrate between these mechanisms, and the authors have acknowledged this already, with the claims of the paper being commensurate to the strength of the findings.

>> We are glad that the revised manuscript is now perceived as a fair characterization of the results.

Reviewer #2: After this second round of revision, I feel that the manuscript has further improved, and gives a fair account of the data and modelling exercise. I again commend the authors for their constructive involvement in this revision exercise. Nonetheless, and despite the fact that I usually try my best not to burden authors unnecessarily, I still feel that some sections need further clarification.

>> We likewise thank the reviewer for their constructive comments throughout the revision process. We hope that the present revision addresses the reviewer's remaining comments.

Especially, I am having a hard time understanding precisely what was done in the parameter recovery and model identification exercise:

Regarding parameter recovery, for instance, I don't understand how the confusion matrices displayed in Supplementary Figure S7 can depict correlations between some parameters and beta or gamma, given that it seems that those should be fixed (by column or line, respectively). I'm also not sure to understand how exactly the parameters were varied (lines 680-683 and lines 709-714).

>> We understand the source of confusion with respect to the parameter recovery analysis. The idea of separating the confusion matrices by levels of β and γ is that both parameters can have a substantial influence on the quality of the parameter recovery matrices. E.g., when testing parameter recovery for α_r (i.e., systematically varying α_r), it makes a difference whether β is set to 0.1 or to 1.6. The columns/rows thus indicate to what value β/γ were set respectively for the construction of a matrix. The exception is of course when β/γ were themselves varied. In these cases, the β/γ values indicated in the columns/rows do not apply. As a consequence, the correlation coefficients for β/γ in each row/column are internal replications of the same analysis, as β/γ was swept through the entire value range independent of what the β/γ value for that column/row indicates.

We now clarify this in the text (see the adapted description of the parameter recovery analysis below), but also in the legend of Figure S7:

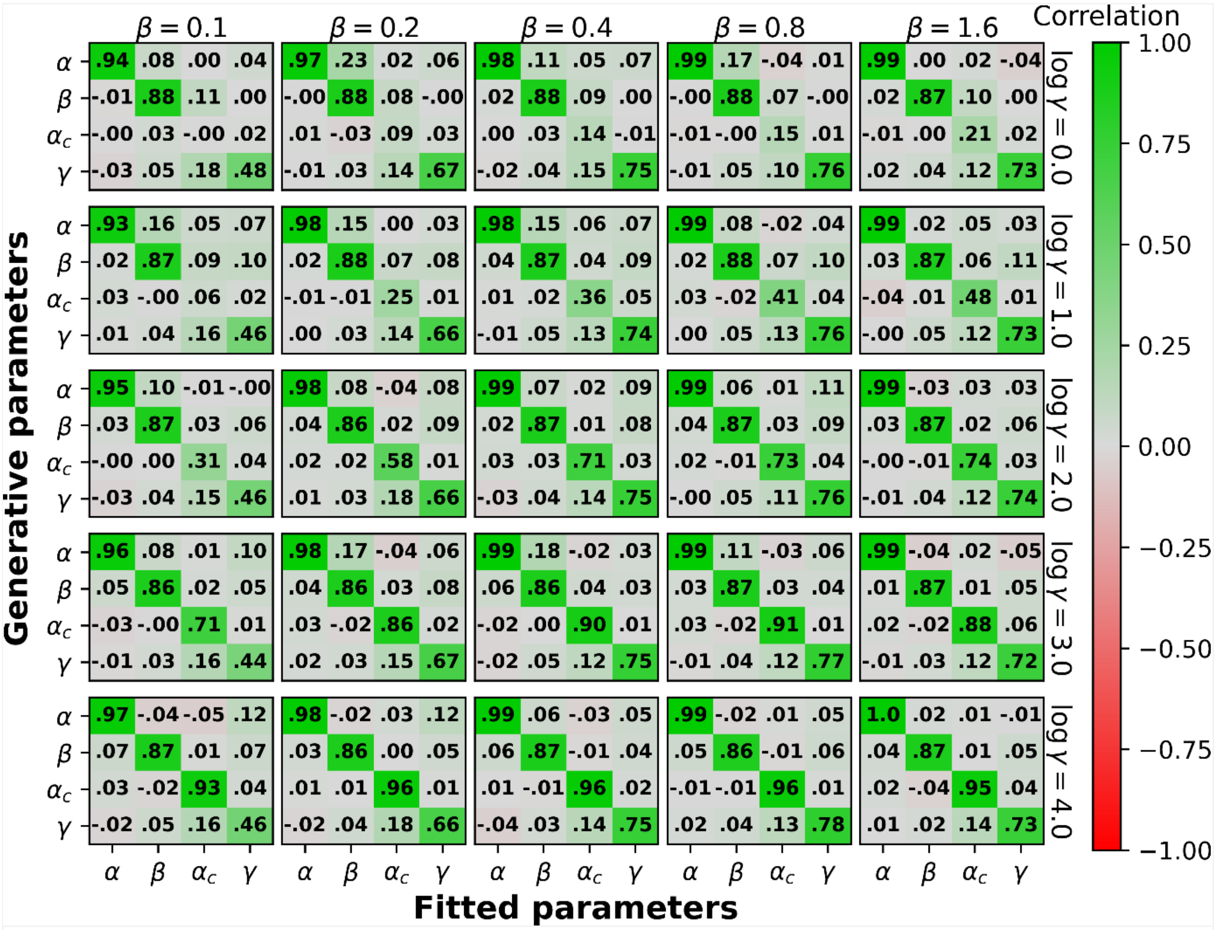

Supplementary Figure S7. Parameter recovery. Pearson correlation matrices between generative parameters and fitted parameters in dependence of different settings for β and γ. The fixed β and γ values provided in the figure thus indicate the parameter values that were used for data generation in the construction of a recovery matrix. An exception is when β and γ were themselves varied – in these cases, the indicated values for β and γ do not apply; instead, different columns constitute internal replications for the recovery of β, and different rows constitute internal replications for the recovery of γ.

In addition, as rightfully requested by the reviewer, we now describe the variation of parameters in more detail, both for the model recovery and the parameter recovery analysis.

Model recovery analysis (previously lines 680-683, now lines 680-688):

> "For each model we simulated choice and confidence data for a range of different parameter settings. k^N different parameter configurations were implemented, where N is the number of parameters in each model (see Figure 4) and k is the number of different values implemented for each parameter ($\alpha_r/\alpha_n/\alpha_c$: range 0.1-1, equidistant steps, k=5; β: range 0.1-1.6, doubling steps, k=5; γ: range 1-100, exponential steps, k=5; λ: range 0.5-5, exponential steps, k=5; η: range -1.5-1.5, equidistant steps, k=6). For each model and parameter combination, we simulated 250 datasets (i.e. subjects). Experimental designs for each dataset were randomly sampled from the design generation function that was used in the behavioral experiment."

Parameter recovery analysis (previously lines 709-714, now lines 720-732):

> "To assess the quality of parameter recovery for the winning model *ConfUnspec*, we generated datasets (i.e. subjects) for which we systematically varied each model parameter with 250 equidistant values between sensible lower and upper bounds (α_r: range 0.01-1; β: range 0.02-2; α_c: range 0-1; γ: range 0-10). As in the model recovery analysis, experimental designs for each dataset were randomly sampled from the design generation function that was used in the behavioral experiment. Parameter recovery correlation matrices were constructed by correlating each varied generative parameter to all fitted parameters. To make the analysis robust against the specific settings of the respective other parameters, we performed this process for each node of the coarse parameter grid of size k^N described above. For example, if the parameters of the coarse parameter grid were ($\alpha_r = 0.1$, $\beta = 0.4$, $\alpha_c = 0.1$, $\gamma = 1$), these exact parameters were used in the data generation for the construction of a correlation matrix, except for the parameter that was systematically varied for the estimation of a specific row in the correlation matrix."

Regarding model identification, how was "the probability that datasets generated with a given model X were best fitted by a model Y" assessed ? Is it a frequency measure, based on a simple AIC comparison between models ? Or something else ? Also, what exactly is referred to as a dataset in those cases ? A subject or a sample of subject equivalent to the study's sample size ?

>> Model recovery was indeed based on the relative frequency of superior AIC fit between models. Thus, p(fit=Y|gen=X) indicates the frequency with which datasets generated with a given model X were best fitted by a model Y. The probability p(gen=X|fit=Y) on the other hand denotes the frequency with which datasets best fitted by a given model Y were generated by a model X. We now clarified and expanded the section explaining this analysis (lines 689ff.):

> "Model recovery was quantified by the probability that datasets generated with a given model X were best fitted by a model Y, p(fit=Y|gen=X), as well as the reverse probability that datasets best fitted by a given model Y were generated by a model X, p(gen=X|fit=Y). To obtain p(fit=Y|gen=X), for all datasets created with a given generative model X, we computed the frequency with which model Y had the lowest AIC value among the competing models (each model was fitted to each dataset). Conversely, to obtain p(gen=X|fit=Y), for all datasets that were best-fitted by model Y (i.e. lowest AIC value), we computed the frequency with which datasets were generated by model X. For p(gen=X|fit=Y), we made sure that the base rate was identical across models, i.e. that the number of datasets generated was equal for all models despite differences in the number of model parameters (and thus in the number of combinatorial parameter settings).
>
> Supplementary Figure S5 shows the results of the model recovery analysis, expanded for different values of α_r and β, the two parameters that are common to all models. Overall, we find excellent model recovery. More precisely, p(fit|gen) is generally much higher when the generative and fitting model are identical relative to when they are different. Perhaps even more importantly, the reverse probability matrix, p(gen|fit), likewise demonstrates good model identifiability (Supplementary Figure S6). That is, datasets that are best fitted by a given model are, in all likelihood, also generated by this model.[…]"

Finally, we now clarify that simulated datasets refer to simulated individual subjects (cf. “datasets (i.e. subjects)” in line 686 and line 721).

So I would like to encourage the author to comprehensively re-write these sections of the methods, taking the time (and space) to describe all steps and choices that have been made, and keeping in mind that reader should understand precisely what has been done, to the point of being able to reproduce the said control analyses.
